# Candidate Gene and Genome-Wide Association Studies for Circulating Leptin Levels Reveal Population and Sex-Specific Associations in High Cardiovascular Risk Mediterranean Subjects

**DOI:** 10.3390/nu11112751

**Published:** 2019-11-13

**Authors:** Carolina Ortega-Azorín, Oscar Coltell, Eva M. Asensio, Jose V. Sorlí, José I. González, Olga Portolés, Carmen Saiz, Ramon Estruch, Judith B Ramírez-Sabio, Alejandro Pérez-Fidalgo, Jose M Ordovas, Dolores Corella

**Affiliations:** 1 Department of Preventive Medicine and Public Health, School of Medicine, University of Valencia, 46010 Valencia, Spain; carolina.ortega@uv.es (C.O.-A.); eva.m.asensio@uv.es (E.M.A.); jose.sorli@uv.es (J.V.S.); Ignacio.Glez-Arraez@uv.es (J.I.G.); Olga.Portoles@uv.es (O.P.); carmen.saiz@uv.es (C.S.); j.alejandro.perez@uv.es (A.P.-F.); 2 CIBER Fisiopatología de la Obesidad y Nutrición, Instituto de Salud Carlos III, 28029 Madrid, Spain; oscar.coltell@uji.es (O.C.); RESTRUCH@clinic.cat (R.E.); 3 Department of Computer Languages and Systems, Universitat Jaume I, 12071 Castellón, Spain; 4 Department of Internal Medicine, Hospital Clinic, Institut d’Investigació Biomèdica August Pi i Sunyer (IDIBAPS), University of Barcelona, Villarroel, 170, 08036 Barcelona, Spain; 5 Oncology Department, Sagunto Hospital, 46250 Sagunto, Spain; jbramire@uv.es; 6 CIBER Cáncer, Instituto de Salud Carlos III, 28029 Madrid, Spain; 7 Nutrition and Genomics Laboratory, JM-USDA Human Nutrition Research Center on Aging at Tufts University, Boston, MA 02111, USA; jose.ordovas@tufts.edu; 8 Department of Cardiovascular Epidemiology and Population Genetics, Centro Nacional de Investigaciones Cardiovasculares (CNIC), 28029 Madrid, Spain; 9 IMDEA Alimentación, 28049 Madrid, Spain

**Keywords:** leptin, genetics, leptin receptor, genome-wide association study, obesity, sex, Mediterranean population, heterogeneity, polymorphisms

## Abstract

Leptin is a hormone crucial in the regulation of food intake and body-weight maintenance. However, the genes and gene variants that influence its plasma levels are still not well known. Results of studies investigating polymorphisms in candidate genes have been inconsistent, and, in addition, very few genome-wide association studies (GWAS) have been undertaken. Our aim was to investigate the genes and gene variants most associated with plasma leptin concentrations in a high-cardiovascular-risk Mediterranean population. We measured plasma leptin in 1011 men and women, and analyzed the genetic factors associated using three approaches: (1) Analyzing the single nucleotide polymorphisms (SNPs) reported in a GWAS meta-analysis in other populations (including an SNP in/near each of these LEP, SLC32A1, GCKR, CCNL, COBLL1, and FTO genes); (2) Investigating additional SNPs in/near those genes, also including the RLEP gene; and (3) Undertaking a GWAS to discover new genes. We did not find any statistically significant associations between the previously published SNPs and plasma leptin (Ln) in the whole population adjusting for sex and age. However, on undertaking an extensive screening of other gene variants in those genes to capture a more complete set of SNPs, we found more associations. Outstanding among the findings was the heterogeneity per sex. We detected several statistically significant interaction terms with sex for these SNPs in the candidate genes. The gene most associated with plasma leptin levels was the FTO gene in men (specifically the rs1075440 SNP) and the LEPR in women (specifically the rs12145690 SNP). In the GWAS on the whole population, we found several new associations at the *p* < 1 × 10^−5^ level, among them with the rs245908-CHN2 SNP (*p* = 1.6 × 10^−6^). We also detected a SNP*sex interaction at the GWAS significance level (*p* < 5 × 10^−8^), involving the SLIT3 gene, a gene regulated by estrogens. In conclusion, our study shows that the SNPs selected as relevant for plasma leptin levels in other populations, are not good markers for this Mediterranean population, so supporting those studies claiming a bias when generalizing GWAS results to different populations. These population-specific differences may include not only genetic characteristics, but also age, health status, and the influence of other environmental variables. In addition, we have detected several sex-specific effects. These results suggest that genomic analyses, involving leptin, should be estimated by sex and consider population-specificity for more precise estimations.

## 1. Introduction

Leptin is an adipokine produced by adipocytes that influences food intake, satiety, body weight, and metabolic activity [1]. Sexual dimorphism in leptin has been observed in rodents and humans, with females having more leptin than males [2,3]. Females secrete more leptin than males and this difference in leptin secretion is intensified with obesity [4]. Besides its role in food intake control, leptin has also been implicated in many other processes, including cardiovascular risk [4,5], cancer [6,7], neurodegenerative diseases [8,9], and in inflammation and immunity [10,11]. For all those reasons, it is especially interesting to delve deeper into the molecular bases that may influence plasma leptin concentrations, as, despite the considerable progress made, they are still not fully understood. Moreover, insufficient attention has often been paid to studying sex-specific effects, which may have resulted in several biases in the estimates, hence the need to reconsider those effects.

The leptin gene (LEP), initially called “ob” in mice [12], is located on chromosome 7 in humans (7q32.1) and consists of three exons [13,14]. Although several low-frequency mutations associated with very low leptin levels, and extreme obesity phenotypes [15,16] have been identified, the effect of common polymorphisms on circulating leptin levels and obesity phenotypes is less clear and inconsistent [17,18,19,20,21,22]. This is because regulation is highly complex and it is known that other genes may also influence leptin levels, as well as on the effect of leptin on obesity and related diseases [23]. Among those genes, the most studied one is the so-called leptin receptor gene (LEPR). Leptin binds to a membrane protein, the leptin receptor that is a class I cytokine receptor. Just as with the leptin gene, several low-frequency variants have been reported in the LEPR gene that result in LEPR deficiency and are associated with severe early-onset obesity and hyperphagia [24,25]. Likewise, common polymorphisms in the LEPR gene have been less consistently associated with plasma leptin levels and obesity phenotypes, depending on the type of population analyzed [26,27,28,29]. In many studies, the polymorphisms in the LEP and LEPR genes are analyzed jointly [30,31,32,33,34].

Other candidate genes that may influence plasma leptin levels have also been identified [35], and genome-wide association studies (GWAS) provide an efficient tool for revealing the main genes associated with this phenotype. However, despite the advantages of this approach, there have been very few GWAS published that have analyzed circulating leptin [36,37]. Outstanding among those is the GWAS and GWAS meta-analysis carried out by Kilpeläin et al. [37], who undertook a two-stage study. In Stage 1, they carried out a GWAS of circulating leptin levels from 32,161 individuals (including 23 studies). In Stage 2, they then tested the loci with *p* < 1 × 10^−6^, in 19,979 additional individuals. Finally, in the joint meta-analysis for men and women, they identified five SNPs reaching genome-wide significance (*p* < 5 × 10^−8^). These SNPs were in/near the following genes: LEP, SLC32A1 (solute carrier family 32 member 1), GCKR (glucokinase regulatory protein), CCNL1 (cyclin-L1), and FTO (fat mass and obesity-associated). In addition to these five robustly associated SNPs, the authors also included the COBLL1 (cordon-bleu WH2 repeat protein like 1) gene to the list of relevant leptin-associated loci from the GWAS. The SNP in this gene was close to genome-wide significance, but previous GWAS studies reported strong associations between this locus and obesity-related measures.

However, it is becoming increasingly important to undertake GWAS on different populations, as the most relevant SNPs in one population may not be the most important in another and so give rise to bias [38]. Likewise, the genetic risk scores (GRS) derived from the top-ranked SNPs identified in a GWAS undertaken on certain populations may not be useful in other populations not included or little represented in the initial GWAS [39]. The Spanish Mediterranean population is, in general, little represented in GWAS, including those carried on European populations, and so specific studies must be undertaken on that population before directly using the SNPs or GRS derived from those GWAS. Specifically, in the case of leptin levels, various studies on different populations [40,41] are using the SNPs reported in the Kilpeläin et al. [37] GWAS as plasma leptin level proxies for Mendelian randomization studies. Therefore, the aims of our study for the whole population and by sex are as follows: (1) to estimate the association between the SNPs reported as most relevant (in/near the LEP, SLC32A1, GCKR, CCNL, COBLL1, and FTO genes) in the Kilpeläin et al. [37] GWAS and leptin levels in a Mediterranean population; (2) to investigate whether other SNPs (after the whole gene-screening) in the 6 genes reported by Kilpeläin et al. [37], plus one that we have added—the LEPR gene—are more highly associated with leptin levels in this population; and (3) to undertake a new GWAS for plasma leptin levels in this Mediterranean population.

## 2. Materials and Methods

### 2.1. Study Design and Participants

We have carried out a cross-sectional analysis at baseline in a Spanish Mediterranean population consisting of participants recruited at the PREDIMED (Prevención con Dieta Mediterránea)-Valencia field center [42], one of the centers participating in the PREDIMED multicenter study [43]. The Valencia field center is located on the East Mediterranean coast of Spain. All participants whose plasma leptin levels were determined at baseline were analyzed (*n* = 1011, including 366 men and 645 women). Eligible subjects were community-dwelling people (55–80 years of age for men; 60–80 years of age for women) who fulfilled at least one of two criteria: type-2 diabetes; 3 or more cardiovascular risk factors: current smoking, hypertension (blood pressure ≥140/90 mmHg or treatment with antihypertensive drugs), low-density lipoprotein cholesterol (LDL-C) ≥160 mg/dL (or treatment with hypolipidemic drugs), high-density lipoprotein cholesterol (HDL-C) ≤40 mg/dL, body mass index (BMI) ≥ 25 kg/m^2^, or a family history of premature cardiovascular diseases [43]. The Institutional Review Board of the Valencia University approved the study protocol (ethical approval code H1422226460525), and all participants provided written informed consent.

### 2.2. Demographic, Clinical, Anthropometric, and Lifestyle Characteristics of the Participants

In the baseline examination, we assessed socio-demographic factors, cardiovascular risk factors, medications and lifestyle variables by validated questionnaires as previously reported [42]. Blood pressure was measured by trained personnel using a validated semi-automatic oscillometer (Omron HEM-70CP; Hoofddorp, the Netherlands) with the subject seated. Weight and height were measured with light clothing and no shoes with calibrated scales and a wall-mounted stadiometer, respectively. BMI was calculated as the weight (in kg) divided by the height (in m^2^). Obesity was defined as a BMI ≥ 30 kg/m^2^. Adherence to the Mediterranean diet was measured by a validated 14-item questionnaire [44]. Physical activity was estimated by the validated Minnesota Leisure-Time Physical Activity questionnaire as previously reported [43].

### 2.3. Biochemical Determinations and Plasma Leptin Measurements

Fasting blood samples were obtained for each participant and stored at −80 °C until biochemical analyses. Fasting glucose, total cholesterol, triglycerides, HDL-C, and LDL-C were determined by standard methods as previously reported [42]. Plasma leptin levels were determined by a sandwich enzyme-linked immunosorbent assay (ELISA) using the Human Leptin ELISA kit, Clinical Range (BioVendor Research and Diagnostic Products, Modrice, Czech Republic), according to the manufacturer’s instructions. Absorbance was measured at 450 nm using the Multiskan EX spectrophotometer (Thermo Electron Corporation, Milford, CT, USA) and the Ascent Software program (Thermo Labsystems, Vantaa, Finland). All determinations were made in duplicate. Results for plasma leptin levels were expressed as ng/mL. The intra- and inter-assay coefficients of variations were <6%.

### 2.4. DNA Isolation and Genome-Wide Genotyping

Genomic DNA was extracted from the buffy coat with the MagNaPure LC DNA Isolation kit (ROCHE Diagnostics). The quantity of double-stranded DNA was measured using PicoGreen (Invitrogen Corporation, Carlsbad, CA, USA). 45 DNA samples did not pass the quality control for genome-wide analysis. Genome-wide genotyping was performed at the University of Valencia using the Infinium OmniExpress-24 BeadChip genotyping array (v1.0 and v1.1) (Illumina Inc., San Diego, CA, USA), according to the manufacturer’s protocol with appropriate quality standards. This array captures approximately 720,000 markers (the number varies depending on the version used: 730,000 for v.1.0 and 716,000 for v.1.1), and we used the 699,221 markers that are common to both versions of the array (v1.0 and v.1.1). Allele detection and genotype calling were performed in the GenomeStudio genotyping module (Illumina, Inc.). Data cleaning was performed by the Department of Computer Languages and Systems of the University Jaume I using standard analysis pipelines implemented in the Phyton programing language combined with the PLINK [45,46]. We filtered out the SNPs not mapped on autosomal chromosomes. In addition, SNPs with a minor allele frequency (MAF) <0.01 or those that deviated from expected Hardy–Weinberg equilibrium (*p* < 1.0 × 10^−4^) were removed. 628,376 SNPs passed the quality filter and remained for further analysis. For our first aim, the genotyping of the polymorphisms reported by Kilpeläin et al. [37]: rs10487505-LEP, rs6071166-SLC32A, rs780093-GCKR, rs900400-CCNL1, rs6738627-COBLL1, and rs8043757-FTO; or their proxies (r^2^ > 0.8), were obtained using the Human Infinium OmniExpress array. The corresponding genotype data were extracted by a Phyton script, created by the authors. Likewise, the screening for all the variants in/near the LEP, SLC32A1, GCKR, CCNL, COBLL1, and FTO genes—plus the LEPR gene—was carried out by extracting their available genotypes in the Infinium OmniExpress-24 BeadChip genotyping array, using another Phyton script, created by the authors. For this procedure, we first used the batch query resource SNP Report with URL: https://www.ncbi.nlm.nih.gov/SNP/snp_ref.cgi?locusId and provided the target gene-ID. The dbSNP-gene type file is used as input argument in the *.bat file for running the Python script. Finally, the Python script produces the corresponding PLINK *.ped and *.map file of genotypes for statistical testing.

### 2.5. Statistical Analysis

Chi-square tests were used to compare proportions. Student’s *t*-tests and ANOVA tests were applied to compare crude means of continuous variables. Leptin and triglyceride levels were log-transformed (ln) for the statistical analyses. To calculate the association between the polymorphisms in the candidate genes reported in the meta-analysis published by Kilpeläin et al. [37] and the plasma leptin levels in our population, general linear models were used. We used the same statistical approach as those authors [37]. After undertaking the log transformation of leptin levels, crude and multivariate models were fitted. Kilpelain et al. [37] first adjusted the models for sex and age. Secondly, to identify loci associated with circulating leptin levels independently of adiposity, they additionally adjusted the models for BMI. Proxies were obtained with the LDlink tool, a web-based application for exploring population-specific haplotypes and correlated alleles [47]. An additive dosage model (alleles 0, 1, and 2) was fitted for each SNP. Firstly, an unadjusted model (Model 1) was used, and then they were adjusted sequentially for age and sex (Model 2) and BMI (Model 3). Additional adjustment for potential confounders, such as diabetes, was carried out when indicated. The estimates were made for both the whole population (men and women jointly) and stratified by sex. The statistical significance of the SNP*sex interaction terms was calculated to assess heterogeneity.

Likewise, for the study of the association between the additional screening of all the SNP available in the Infinium OmniExpress-24 BeadChip genotyping array in/near the LEP, SLC32A1, GCKR, CCNL, COBLL1, and FTO genes [37], and the plasma leptin levels in our population, we also used general linear models as indicated above. Besides those genes [37], we also included the LEPR gene due to its importance in other populations. Unadjusted (Model 1) and models sequentially adjusted for sex and age (Model 2) and BMI (Model 3) were used. Additional adjustments were carried out when indicated. Analyses on the whole population and others stratified by sex were undertaken. The statistical significance of the SNP*sex interaction terms was also calculated. For the candidate gene analyses, all tests were two-tailed and *p*-values < 0.05 were considered statistically significant.

For GWAS, association analyses were carried out using PLINK v1.9 [45,46]. Additive genetic models (0, 1, or 2 copies of the variant allele) were fitted. General linear models were used. Model 1 (unadjusted), Model 2 (adjusted for sex and age), and Model 3 (additionally adjusted for BMI), were estimated. Further adjustments were made when indicated. Coefficients for the minor allele were obtained. These analyses were performed on the whole population and stratified by sex. Moreover, the statistical significance of the genome-wide SNP*sex interaction term was computed. We used the conventional threshold of *p* < 5 × 10^−8^ for genome-wide statistical significance, as well as the standard *p*-value for suggestive genome-wide significance (1 × 10^−5^). As these thresholds are very conservative for a small sample size, SNPs with *p*-values below 5 × 10^−5^ are also shown in the tables so that they may be compared with other studies that have analyzed similar associations and may be included in meta-analyses. SNPs were rank-ordered according to the minimum *p*-value in the corresponding models. Manhattan plots were generated using Haploview software to visualize the results [48]. We used LocusZoom.js to generate locus-specific graphical displays of the position of the selected SNPs in the GWAS to nearby genes and local recombination hotspots [49], as well as to indicate the linkage disequilibrium (LD). LD coefficients between selected SNPs were also estimated with Haploview [48]. Quantile–quantile plots, comparing the expected and observed *p*-values, were performed in the R-statistical environment.

## 3. Results

### 3.1. Characteristics of the Population and Association of Leptin Levels with BMI in Men and Women

Table 1 provides an overview of the demographic, clinical, biochemical, lifestyle characteristics and plasma leptin concentrations of the 1011 subjects according to sex (*n* = 366 men and *n* = 645 women).

**Figure 1:**
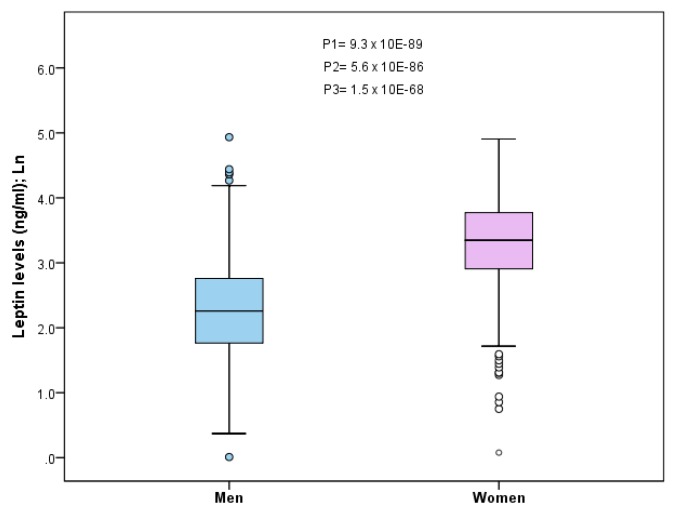
Sex-specific differences in plasma leptin concentration (Ln) in men (*n* = 366) and women (*n* = 645). Unadjusted values in a dotplot by sex are presented, as well as several *p*-values for the sex comparisons after ln-transformation. *p*_1_: age-adjusted; *p*_2_: additionally adjusted for diabetes; *p*_3_: additionally adjusted for diabetes, smoking, adherence to Mediterranean diet, and physical activity.

Participants were high cardiovascular risk subjects aged 55–80 years. Leptin concentrations (unadjusted model) were 2.6 times higher (*p* = 1.7 × 10^−89^) in women (34.3+/−0.9 ng/mL) than in men (13.1+/−0.7 ng/mL). Figure 1 shows the dotplot for plasma leptin concentrations (ln) in men and women. *p*-values for several adjusted models are also presented. The age adjustment did not change the statistical significance (using the ln-transformation) of the differences between men and women (*p*_1_ = 9.3 × 10^−89^). Additional adjustment for diabetes, smoking, adherence to the Mediterranean diet, and physical activity (*p*_3_) slightly reduced the statistical significance of the differences in leptin levels between men and women, but those differences still prevailed (*p*_3_ = 1.5 × 10^−68^).

**Figure 2:**
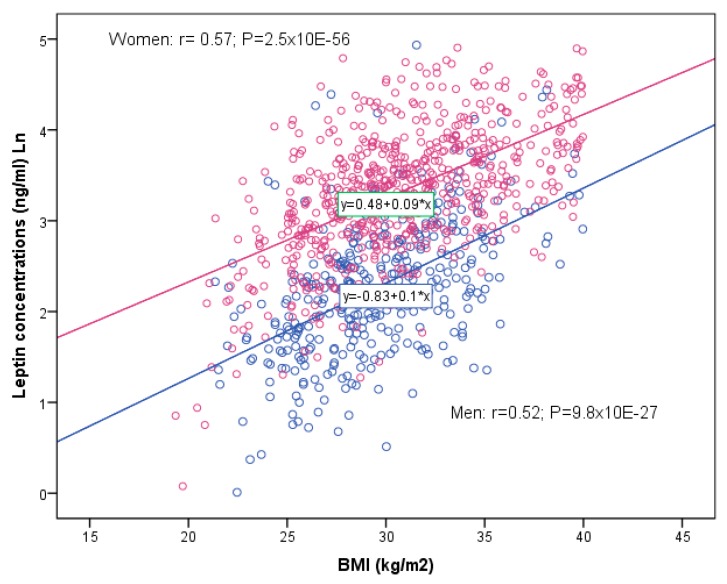
Scatter plot for the association between BMI and plasma leptin concentrations (ln) in men (*n* = 366) and women (*n* = 645). Linear regression coefficients and *p*-values were estimated for men and women respectively.

As expected, plasma leptin levels in our population had a high association with BMI. Figure 2 shows the sex-specific scatter plot and the linear adjustment between plasma leptin levels (Ln) and BMI in both men and women. We observed a highly statistically significant direct correlation between these parameters (r = 0.52; *p* = 9.8 × 10^−27^ in men and r = 0.57; *p* = 2.5 × 10^−56^ in women).

### 3.2. Association between the Selected SNPs in Candidate Genes (LEP, SLC32A1, GCKR, CCNL, COBLL1, and FTO) and Plasma Leptin Concentrations

First we tested the association between plasma leptin levels and the SNP in the candidate genes selected as the most relevant in the GWAS of Kilpeläin et al. [37] for circulating leptin levels: rs10487505-LEP, rs6071166-SLC32A, rs780093-GCKR, rs900400-CCNL1, rs6738627-COBLL1, and rs8043757-FTO; or their proxies (r^2^ > 0.8), when the SNP itself was not in the genotyping array. The following proxies (indicating the LD with the initial SNP), were used: rs2167289-LEP (r^2^ = 0.81), rs6027422-SLC32A (r^2^ = 1.0), rs17451107-CCNL1 (r^2^ = 0.92), rs7609045-COBLL1 (r^2^ = 0.98), and rs17817449-FTO (r^2^ = 1.0). Table 2 shows the results for the whole population (*n* = 966 subjects with valid genome-wide data including 351 men and 615 women), both in the unadjusted model (Model 1) and that adjusted for age and sex (Model 2).

In neither of them is a statistically significant association observed between the candidate SNPs [37] and plasma leptin levels in our Mediterranean population. Neither did the additional adjustment of the results for BMI alter the statistical significance of the associations (Supplemental Table S1). Moreover, an additional adjustment of the models for diabetes did not contribute to increasing the statistical significance of the associations either (not shown). Likewise, additional adjustment for smoking, physical activity, and adherence to Mediterranean diet did not change the statistical significance (not shown). We then carried out an analysis stratified by men (Table 3) and women (Table 4).

In men, we did not find any association between any of the candidate SNPs and plasma leptin levels in any of the models studied. However, in women, we did find several associations. In the crude model (Model 1), the SNP rs17451107-CCNL1 was significantly associated with lower leptin levels in carriers of the minor allele (B = −0.085 +/−0.041; *p* = 0.038). In addition, the rs780093 SNP in the GCKR gene was significantly associated with leptin levels in the crude model (*p* = 0.049), with lower leptin levels in carriers of the minor allele. Nevertheless, on adjusting for age, the rs78009-GCKR SNP lost statistical significance.

The additional adjustment for BMI (Supplemental Table S2) did not take statistical significance away from the rs17451107-CCNL1, which remained associated with plasma leptin levels even after a later adjustment in model 3 for diabetes (*p* = 0.0014, not shown).

These results not only indicate some heterogeneity per sex, but also that the characteristics of this population (either of geographic origin or being a high cardiovascular risk) are important if the results of the most important SNP associations reported in the meta-analysis of Kilpeläin et al.’s GWAS [37] are not replicated. We, therefore, decided to investigate whether other SNPs in the same candidate genes could be more associated with leptin levels and better reflect the genetic characteristics of this population.

### 3.3. Screening of SNPs in the LEP, SLC32A1, GCKR, CCNL, COBLL1, FTO, and LEPR Genes for Investigating Their Association with Plasma Leptin Levels

We obtained the genotype of all the SNPs in/near the LEP, SLC32A1, GCKR, CCNL, COBLL1, FTO, and LEPR genes that were included in the Human OmniExpress array, and their associations with plasma leptin levels in the whole populations were investigated. Table 5 shows the SNP, MAF, beta, and *p*-values for the association of the top-ranked SNPs in the whole population (*n* = 966). Top-ranked SNPs were listed three times in accordance with each statistical model (Model 1, Model 2, and Model 3). We can observe that, in this population, the SNPs in the LEPR have a highly significant association with leptin levels, both in the crude model and in the models adjusted for age and sex, the rs10749753-LEPR SNP (MAF: 0.30) being the one that presented the highest statistical significance (*p* = 0.016) in the whole population, with the minor allele related with higher leptin levels. Even after adjustment for BMI, that SNP maintained its statistical significance (*p* = 0.001). Some SNPs in the FTO gene were also shown to be associated with plasma leptin levels (i.e., rs1075440-FTO; *p* = 0.017 or rs17819063-FTO; *p* = 0.031), and unlike what happens in the GWAS of Kilpeläin et al. [37], several SNPs in the FTO gene do not lose their statistical significance in their association with leptin, even after adjustment for BMI (i.e., rs1075440-FTO; *p* = 0.037 or rs17819063-FTO; *p* = 0.016). Further adjustment for diabetes did not change the statistical significance of the associations.

On analyzing the SNP*sex interaction determining plasma leptin levels, we found several statistically significant interaction terms (Supplemental Table S3). The most significant SNP*sex interactions were with the rs9436297-LEPR and rs1362570-FTO SNPs (*p*-interaction SNP*sex = 0.003 and *p*-interaction = 0.010, respectively). That shows these polymorphisms to be heterogeneous per sex in their association with leptin levels. On stratifying the association of the SNPs with leptin levels for men (Table 6) and women (Table 7), we observed that the SNPs that presented the greatest associations with leptin in men are those found in the FTO gene (the rs1075440-FTO being the top-ranked after adjustment for age and BMI; *p* = 0.010), whereas in women it was mainly the LEPR gene (the rs12145690-LEPR was the top-ranked after adjustment of age and BMI; *p* < 0.001).

Some of these SNPs were in LD, but not others. Supplemental Figure S1 shows the LD plots (r^2^ values) for the FTO (panel A) and the LEPR (B) statistically significant SNPs.

### 3.4. GWAS for Plasma Leptin Concentrations in This Mediterranean Population

Finally, we undertook a GWAS in order to investigate which SNPs at the genome-wide level were more significantly associated with plasma leptin levels in this population. Supplemental Figure S2 shows the corresponding Q–Q plot for this GWAS. Table 8 shows the results obtained for the top-ranked SNPs, in the model adjusted for age and sex for the whole population and Supplemental Table S4 shows the results of the model additionally adjusted for BMI. Additional adjustment for diabetes did not change the statistical significance of the associations (not shown). No SNP reached statistical significance at the GWAS level (*p* < 5 × 10^−8^), a foreseeable result bearing in mind the low level of statistical significance achieved in the prior meta-analysis of thousands of participants. Various SNPs, however, did achieve a statistical significance of *p* < 1 × 10^−5^, suggestive of an association at the GWAS level that would require confirmation in other studies.

**Figure 3:**
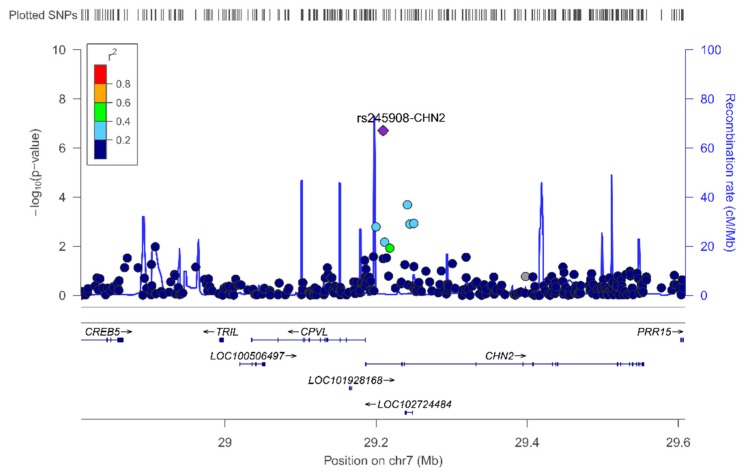
Regional plot for the top-ranked SNP rs245908, located in the CHN2, on chromosome 7. Results in the whole population.

The top-ranked SNP in the unadjusted model (Model 1) was rs245908 (*p* = 1.95 × 10^−7^), located in the CHN2 (Chimerin 2) gene (Figure 3). After adjustment for sex and age, the top-ranked SNP was the rs10737381 located at LOC105378641, on chromosome 1 (Supplemental Figure S3). The second top-ranked SNP was again the rs245908-CHN2. The CHN2 gene is located on chromosome 7 and encodes a guanosine triphosphate (GTP)-metabolizing protein.

**Figure 4:**
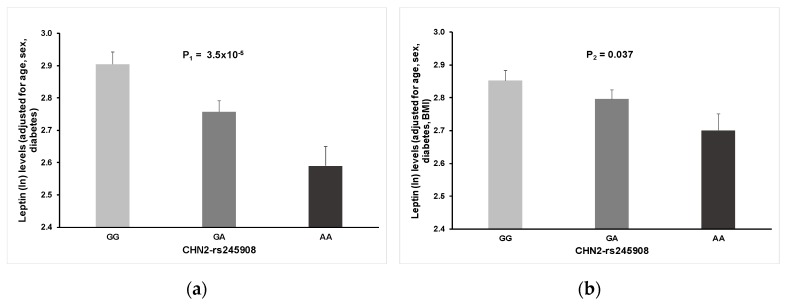
Associations between the rs245908- CHN2 SNP and plasma leptin concentrations (ln) in the whole population: (**a**) Means adjusted for sex, age, and diabetes; (**b**) Additional adjustment for BMI. *p*-values for statistical differences among genotypes (*n* = 382 GG, 444 GA, and 138 AA) were estimated in the corresponding adjusted model. Error bars: SE.

Although no previous works link this gene with plasma leptin concentrations, there are prior known associations with obesity and diabetes [50,51]. The minor allele of this gene was associated with lower plasma leptin concentrations. Supplemental Figure S4 shows the dotplot for plasma leptin concentrations (ln) depending on the CHN2 SNP (unadjusted values). Figure 4 shows the adjusted means of plasma leptin concentrations in the whole population after adjustment for sex, age, and diabetes (A), and additionally adjusted for BMI (B). Even additional adjustment for BMI, the rs245908-CHN2 remained significantly adjusted with leptin levels. Results were similar in men and women (not shown).

**Figure 5:**
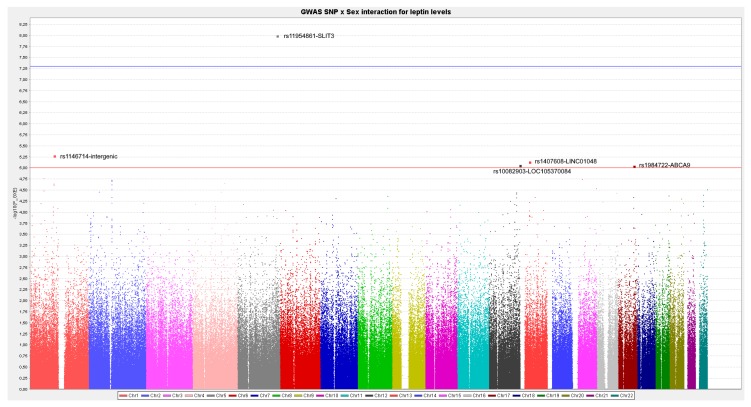
Manhattan plot for the GWAs results for the Gene–sex interactions between SNPs and sex in determining plasma leptin concentrations (ln) in the whole population.

When we tested the SNP*sex interactions at the genome-wide level in determining leptin levels we obtained a statistically significant interaction at the GWAS level, involving the SLIT3 (Slit Guidance Ligand 3) gene as well as some SNP*sex interactions at the suggestive GWAS level. Figure 5 shows the corresponding Manhattan plot showing the *p*-values for these interactions as well as the SNP having *p*-values for the interaction term with sex <1.0 × 10^−5^. The top-ranked SNP for the interaction with sex was the rs11954861-SLIT3 (*p* = 1.1 × 10^−8^) and was located on chromosome 5. Supplemental Figure S5 shows the regional plot. The second top-ranked SNP for the sex-SNP interactions (*p* = 5.2 × 10^−6^) was the rs1146714 (intergenic), located on chromosome 1 (Supplemental Figure S6).

Table 9 shows the top-ranked *p*-values for the SNP*sex interaction terms, as well as the effect (beta) for plasma leptin concentrations in men and women. The minor allele for the rs11954861-SLIT3 SNP was associated with higher plasma leptin levels in men, but with a decrease in women. Although some previous investigations have reported sex-specific effects for the SLIT3 gene, including its differential gene expression regulated by estrogens [52] as well as for the associations between the SLIT3 gene and several parameters [53,54], this is the first time that SLIT3-sex-specific differences in plasma leptin have been found. Likewise, for the other top-ranked genes, we suggest sex-SNP interactions (Table 9).

**Figure 6:**
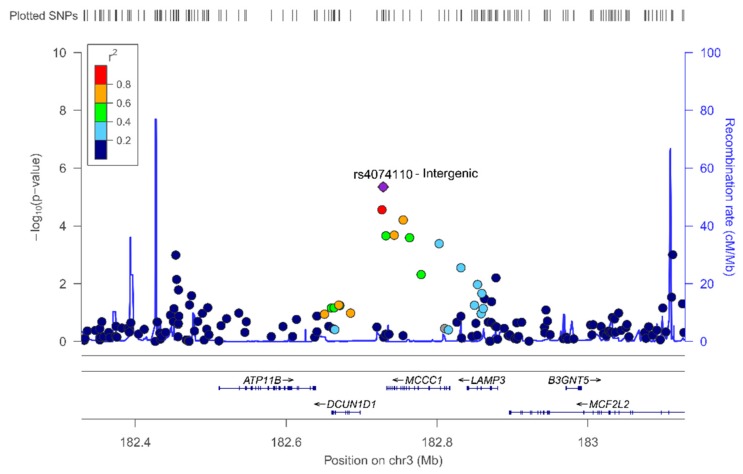
Regional plot for the top-ranked SNP rs4074110 (intergenic), on chromosome 3. Results in men.

Our findings are novel and more research in characterizing these interactions has to be done. Considering the scarcity of data from sex-specific GWAS in determining plasma leptin concentrations, we present the association results including the top-ranked SNPs in men (Supplemental Table S5) and women (Supplemental Table S6) for further comparisons in subsequent studies. In men, the top-ranked SNP both in the unadjusted and in the model adjusted for sex, was the rs-4074110 (intergenic) at *p* < 1 × 10^−5^ (Figure 6 shows the regional plot). The minor allele was associated with higher plasma leptin concentrations.

Our associations for this SNP are novel however, as we detected the rs607437 in the SORCS1 (sortilin-related VPS10 domain containing receptor 1) gene (*p* = 5.6 × 10^−6^) as the second top-ranked SNP in men. This gene is expressed in neurons of the arcuate nucleus of the hypothalamus control and controls energy balance [55]. Moreover, it has been widely studied in leptin-deficient obese mice and linked to insulin resistance and diabetes [56], as well as to neurodegeneration in both mice and humans [57]. However, after adjustment for BMI, the statistical significance was attenuated. Among the top-ranked SNP after BMI adjustment in men, we detected the rs-4361832-NTRK2 (neurotrophic receptor tyrosine kinase 2) associated with plasma leptin concentration. They are consistent in humans and mice showing that the NTRK2 gene plays a critical role in the control of energy balance and obesity [58]. Furthermore, Nkx2.1-Ntrk2-/- mice show increased blood glucose, serum insulin and leptin levels [59], so providing mechanistic support for our GWAS findings.

**Figure 7:**
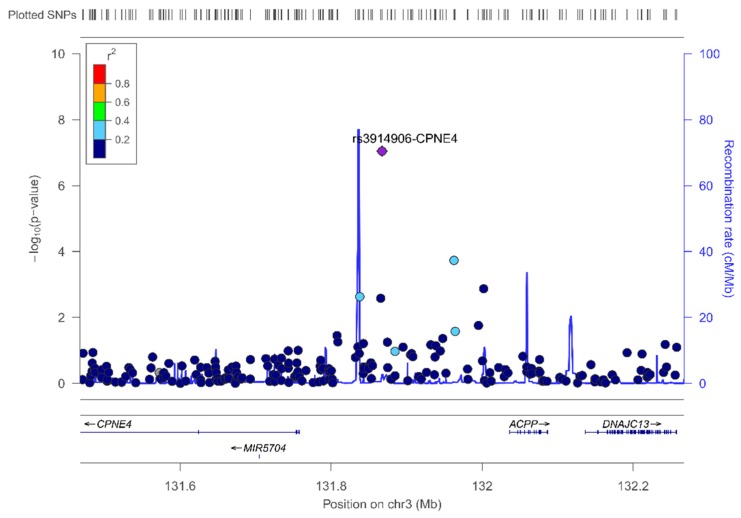
Regional plot for the top-ranked SNP rs3914906 located at CPNE4, on chromosome 3. Results in women.

In the sex-specific GWAS in women, we did not detect these SNPs as top-ranked. The lead SNP in women was the rs3914096-CPNE4 (copine 4) (Figure 7 shows the regional plot). This gene encodes a calcium-dependent, phospholipid-binding protein involved in membrane trafficking.

Some polymorphisms in the CPNE4 gene have been associated with cardiovascular and neurodegenerative diseases [60,61]. The CPNE4 gene loses its significance after adjustment for BMI. However, in women, after the additional BMI adjustment, the SLIT3 gene was the second top-ranked (*p* = 2.05 × 10^−6^), supporting the gene–sex interaction results.

## 4. Discussion

In this study, we have investigated the SNPs most associated with plasma leptin concentrations in a high cardiovascular risk Mediterranean population. We have shown that there are several sex-specific genetic associations, as well as several population-specific-differences, concerning the central genes and SNPs that are most significantly associated. These results contribute, on one hand, to emphasizing the essential need to study the homogeneity or heterogeneity per sex of genetic associations (in this case, the SNPs associated with leptin levels) in order to obtain fewer biased results; thus contributing to more profound knowledge for future precision medicine [62,63]. On the other hand, these results also underscore the importance of studying specific populations instead of extrapolating results of gene variants obtained in studies undertaken in other populations [38,64,65,66,67]. Several publications have pointed out that, for precision medicine, disease risk is likely to be miscalculated if GWAS results obtained in one population are naively used to compute GRS for a geographically different population [38,66]. It has been pointed out that most GWAS have been undertaken using European ancestry samples [39]; however, even those GWAS have mainly been conducted in populations based in the United States, Germany, England, Finland, Sweden, Denmark, France, Austria, or Italy, among others [39,66,67], and very few GWAS have analyzed the Spanish Mediterranean population. That means that information is unavailable on whether the gene variants that are associated to a higher degree in the GWAS and meta-analysis of GWAS in other countries are also the most relevant for the Spanish Mediterranean population. Previous studies indicate that there may be substantial differences in the predictive value of gene variants depending on different populations [39,66]. In this regard, European north–south gradients have even been described for several polymorphisms and ancestry markers [68,69,70,71,72,73]. These small genetic differences may influence the top-ranked SNPs in the corresponding GWAS, requiring finer tuning for the genetic structure in precision medicine/nutrition. In the specific case of leptin, very few GWAS have been undertaken worldwide, given that it is not a measurement readily available in large epidemiological studies. Outstanding among the few GWAS that have been undertaken is that of Kilpeläin et al. [37], who in stage 1 analyzed plasma leptin including up to 32,161 individuals of European descent from 23 studies (mainly from Europe and the United States), but none of those 23 included a Spanish population and neither did their Stage 2 meta-analysis, which analyzed up to 19,979 additional individuals of European descent from 13 studies. Therefore, it is not surprising that in our Spanish Mediterranean population, the effect of the six SNPs (rs10487505-LEP, rs6071166- SLC32A, rs780093- GCKR, rs900400-CCNL1, rs6738627-COBLL1, and rs8043757-FTO), reported as most significant by Kilpeläin et al. [37], did not reach statistical significance in our whole population. By sex, only one SNP was statistically significant in women after the adjustment for BMI. However, when we carried out, in our Mediterranean population, a whole gene screening to capture a more complete set of SNPs in the same candidate genes (LEP, SLC32A, GCKR, CCNL1, COBLL1, and FTO), we did obtain several statistically significant association between other SNPs in these genes and plasma leptin concentrations. In addition to those genes, we included the LEPR gene that, although not showing up as relevant in the GWAS de Kilpeläin et al. [37], has been reported in other studies as having significant associations of SNPs in the LEPR gene with circulating leptin levels [24,26,33,74]. In the LEPR screening, we also found several SNPs showing statistically significant associations with leptin levels in our population. These results are relevant because they highlight the pitfalls of using SNPs or GRS, derived from the above mentioned GWAS [37], as proxies for plasma leptin concentrations for setting individual-level metrics of genetic risk in Mendelian randomization studies [75], in this, and other populations (i.e., the SNPs obtained in the GWAS undertaken by Kilpeläin et al. [37], have been used in Mendelian randomization studies for the association between leptin and Alzheimer’s disease [40] or bone mineral density [41]).

Another factor that could act independently, or jointly with geographical differences, is population characteristics. In our case, we did not study a general Mediterranean population, but an elderly, high cardiovascular risk population in which there may be factors that also modulate the association between the candidate SNPs [37] and plasma leptin levels. These gene-health status-environment interactions should be additionally analyzed in other studies to better evaluate the genetic contribution. We must underscore that candidate SNPs in some studies may not be the most relevant in other studies with a population of different age, environment, or health status characteristics. This adds then a more dynamic component to the effect of SNPs on a phenotype, in this case, leptin, which may vary with age, sex, and population traits. As an example of this, we could point to the results of Helgeland et al. [76], where they analyzed, at the GWAS level, the associations between the genome and BMI in children at 12 time points, from birth to eight years (9,286 children, more than 70,000 measurements) in a Norwegian cohort. Their results clearly show that the effect of an SNP on BMI is variable and either can show associations with BMI or not, depending on when it is measured. For example, for the SNPs in the LEPR, they identified a transient effect with no effect at birth, increasing effect in infancy, and slight effect after 5 years. A similar transient effect near the leptin gene was identified.

Therefore, in generating results for future precision medicine and nutrition, it will be necessary to pay more attention to the possible dynamic characteristics of SNP associations with the traits that determine them, as well as the characteristics of the populations analyzed. Of relevance will be the analysis of sex-specific effects [62,63,77]. In the case of leptin, its levels are higher in women than in men at all ages (1–5). In our analysis of SNP*sex interactions, we found various SNPs in candidate genes with statistically significant interactions (at the nominal *p*-value) with some LEPR and FTO SNPs. We also found sex-specific effects concerning the candidate genes/SNPs that are more significantly associated with plasma leptin levels. In men, the FTO gene presents the most significant associations, whereas, in women, it was found to be the LEPR gene. Currently, we do not know the potential mechanisms behind these sex-specific differences. Few studies have carried out formal analysis of the gene–sex interactions in determining leptin level and this information is lacking. There have been studies associating polymorphisms in the FTO gene with plasma leptin levels in various populations [37,78,79,80,81,82], concluding that leptin could be a possible intermediary contributing to the association between the FTO polymorphism and adiposity. However, those studies did not properly investigate potential sex-related differences. Therefore, sex-specific mechanisms need to be better studied.

In the GWAS analysis undertaken to reveal the SNPs most associated with plasma leptin levels in the Spanish Mediterranean population, we did not identify any association at the conventional GWAS level (5 × 10^−8^). However, we identified some SNPs associated with plasma leptin levels at the suggestive GWAS level (1 × 10^−5^) in the whole population as well as in men and women. Although our sample size was not large, this being a limitation, the homogeneity in the geographic origin as well as the homogeneity in the population characteristics and the duplicate measurements of leptin concentrations, increased statistical power. Furthermore, we can state that the percentage of variability explained by the top-ranked SNPs is very low. These data are in agreement with other GWAS studies [37] and support the finding that the genetic associations obtained did not reach a great statistical significance, suggesting an important modulation by environmental factors and/or population characteristics. The additional adjustments for BMI in our models have allowed us to identify which SNPs seem to have an effect on leptin regardless of weight and those that are dependent. This adjustment was proposed by Kilpeläinen et al. [37], although they had already stated that one possible limitation may be that BMI is not the best measurement of adiposity. They undertook a secondary analysis in a subsample consisting of 13 studies that had data on both BMI and body fat percentage assessed by dual-energy X-ray absorptiometry or bioimpedance analysis. In that study, they were able to observe that the effects were similar to those adjusted for BMI or adjusting the models for body fat %, so suggesting that adjustment for BMI as compared with a more direct measure of adiposity did not compromise the ability to identify adiposity-independent leptin-associated loci. In our case, we did not have data on body fat % measurements available and so were unable to test the possible differential influence or not.

As far as we know, this is the first GWAS carried out on a Spanish Mediterranean population for the circulating leptin phenotype. Detailed analysis and some discussion of the top-ranked SNPs, both in the whole population and by sex, are provided in the results section (see Results for more information). We have outlined the novelty of our findings regarding the association between the rs245908-CHN2 SNP and plasma leptin concentration. The minor allele of this common variant (MAF: 0.38) is associated with lower leptin concentrations. This association, although attenuated, remained statistically significant after BMI adjustment. Curiously, it was observed for both men and women in the stratified analysis (not shown). Previous studies reported associations between the CHN2 gene and insulin-resistance, diabetes, and obesity phenotypes [50,51,83] as well as with addiction and neurological diseases [84,85,86].

In the sex-stratified GWAS for men and women, we have identified novel sex-specific associations with plasma leptin. Hence, we identified a suggestive male-specific loci at chromosome 3 involving the SNP rs4074110 (intergenic) near the methylcrotonoyl-CoA carboxylase 1 (MCCC1) and defective in cullin neddylation 1 domain containing 1 (DCUN1D1) genes. Likewise, we identified a suggestive female-specific loci at chromosome 3 in the CPNE4 gene (rs3914096). Previous studies have associated this gene with cardiovascular and neurodegenerative phenotypes [60,61], but did not explore plasma leptin concentrations. Although these SNPs did not reach statistical significance at *p* < 5 × 10^−8^, they did reach a suggestive *p*-value (*p* = 3.28 × 10^−6^ for the rs4074110 and *p* = 9.04 × 10^−8^ for the rs3914096), respectively. As GWAS on leptin concentrations are very scarce, the interest in showing these results lies in the fact that they will provide information to compare with other studies and for inclusion in meta-analyses. On analyzing the statistical significance of the gene–sex interaction terms at the genome-wide level, we detected several SNPs having sex-specific effects at *p* < 1 × 10^−5^. Moreover, we detected a SLIT3 gene–sex interaction at the GWAS level of significance (*p* < 5 × 10^−8^). Interestingly, SLIT3 is regulated by estrogens [52]. The SLIT family interacts with the ROBO family of transmembrane receptors in a wide variety of physiological processes. SLIT3 is widely expressed in human tissues and its deregulation has been associated with cancer, including breast cancer [87]. SLIT3 also has been related to several processes in the ovary including follicle development, among others [52,88,89]. This gene presents a higher association with leptin concentrations in women than in men, and in opposite directions for the top-ranked SNP (rs11954861-SLIT3). Although the SLIT3–sex interaction in determining leptin levels is reported here for the first time, several studies have shown other sex-specific effects for this gene. Thus, Park et al. [53], showed that Slit3-KO mice displayed anxiety-like behaviors, and these effects were mainly observed in female KO mice. Likewise, Chung et al. [90], in a genome-wide interaction analysis between sex and SNPs on intracerebral hemorrhage risk, obtained a SNP*sex interaction at the GWAS level with an SNP in the SLIT3 gene (rs2337552; *p* = 1.0 × 10^−8^). In the sex-stratified association tests, in agreement with our results, the effect directions differed between men and women. Additional studies are required to check the consistency of these observations which, together with our study, will contribute to a better understanding of the genetics of leptin and subsequently for obesity-cardiovascular phenotypes and interactions with diet.

## 5. Conclusions

Our results, in a Mediterranean population, show small or lack of association for the most significant SNPs reported in a previous plasma leptin GWAS meta-analysis. However, genetic screening of these genes to capture additional SNPs provided several significant associations with leptin levels. These results highlight the potential problems when extrapolating GWAS results from one population to estimate genetic associations with leptin in another population. These population-specific differences may be wide-ranging. They would include not only genetic characteristics, but also other factors such as age, health status, and the influence of other modulating environmental variables. Moreover, we have detected several sex-specific associations between SNPs and leptin concentrations both at the candidate gene and at the GWAS level, providing novel results and suggesting that the GRS for risk associations and Mendelian randomization studies involving leptin should be estimated by sex and should consider population-specificity for more precise estimations.

## Figures and Tables

**Table 1:** Demographic, clinical, lifestyle, and genetic characteristics of the study participants at baseline according to sex.

	Total (*n* = 1011)	Men (*n* = 366)	Women (*n* = 645)	*p*
Age (years)	66.9 ± 0.2	66.4 ± 0.4	67.3 ± 0.2	0.035
Weight (Kg)	76.4 ± 0.4	81.8 ± 0.6	73.4 ± 0.4	<0.001
BMI (Kg/m^2^)	30.3 ± 0.1	29.6 ± 0.2	30.7 ± 0.2	<0.001
Waist circumference (cm)	102.6 ± 0.4	104.7 ± 0.6	101.5 ± 0.5	<0.001
SBP (mm Hg)	146.8 ± 0.6	148.4 ± 1.0	145.9 ± 0.8	0.065
DBP (mm Hg)	81.9 ± 0.3	82.6 ± 0.6	81.5 ± 0.4	0.105
Total cholesterol (mg/dL)	208.1 ± 1.2	199.6 ± 1.9	212.8 ± 1.6	<0.001
LDL-C (mg/dL)	129.0 ± 1.1	124.3 ± 1.8	131.6 ± 1.4	0.002
HDL-C (mg/dL)	52.7 ± 0.4	48.0 ± 0.6	55.4 ± 0.5	<0.001
Triglycerides ^1^ (mg/dL)	132.9 ± 2.6	139.0 ± 4.4	129.6 ± 3.2	0.052
Fasting glucose (mg/dL)	119.7 ± 1.2	127.4 ± 2.2	115.4 ± 1.5	<0.001
Leptin^1^ (ng/mL)	26.62 ± 0.71	13.10 ± 0.70	34.29 ± 0.92	<0.001
Type 2 diabetes: n, %	470 (46.5)	201 (54.9)	269 (41.7)	<0.001
Obesity: n, %	508(50.2)	162(44.3)	346(53.6)	<0.001
Current smokers: n, %	122(12.1)	93(25.4)	29(4.5)	<0.001
Non-drinkers: n, %	442(43.7)	86(23.5)	356(55.2)	<0.001
Physical Activity (MET-min/day)	169 ± 5	220 ± 12	130 ± 5	<0.001
Adherence to MedDiet (P14) ^2^	8.40 ± 0.07	8.55 ± 0.11	8.36 ± 0.08	0.159

Values are mean ± SE for continuous variables and number (%) for categorical variables. BMI indicates body mass index. SBP: systolic blood pressure. DBP: diastolic blood pressure. MedDiet, Mediterranean diet. P: *p*-value for the comparisons (means or %) between men and women. MET: metabolic equivalent. ^1^ Triglycerides and leptin were ln-transformed for statistical testing. ^2^ Quantitative 14-item questionnaire for adherence to Mediterranean diet.

**Table 2:** Association of candidate SNPs with plasma leptin concentrations (ln) in men and women.

				Unadjusted ^3^			Adjusted ^4^	
Chr	Gene	SNP ^1^	SNP/Proxy ^2^	Beta ^3^	SE ^3^	P ^3^	Beta ^4^	P ^4^
7	LEP	rs10487505	rs2167289	0.0120	0.0403	0.767	−0.0124	0.707
2	GCKR	rs780093	rs780093	−0.0074	0.0396	0.851	−0.0425	0.190
3	CCNL1	rs900400	rs17451107	−0.0007	0.0418	0.987	−0.0374	0.275
20	SLC32A1	rs6071166	rs6027422	0.0609	0.0422	0.150	0.0540	0.118
2	COBLL1	rs6738627	rs7609045	0.0587	0.0416	0.158	0.0399	0.241
16	FTO	rs8043757	rs17817449	0.0025	0.0401	0.951	0.0080	0.807

Chr: chromosome. ^1^ Single nucleotide polymorphism (SNP)s reported by Kilpeläin et al. [37]. ^2^ Tested SNPs in this population (*n* = 966 men and women). These SNPs are original SNP or proxies (r^2^ > 0.8). ^3^ Model 1, unadjusted general linear model (GLM). SNPs were tested in an additive model (0, 1, or 2 minor alleles). ^4^ Model 2, GLM adjusted for sex and age. Beta: indicates the regression coefficients per one minor allele. SE: standard error of Beta. P: *p*-value.

**Table 3:** Association of candidate SNPs with plasma leptin concentrations (ln) in men.

				Unadjusted ^2^			Adjusted ^3^
Chr	Gene	SNP ^1^	MAF	Beta ^2^	P ^2^	Beta ^3^	P ^3^
7	LEP	rs2167289	0.36	0.0876	0.115	0.0867	0.1183
2	GCKR	rs780093	0.29	0.0218	0.696	0.0196	0.7260
3	CCNL1	rs17451107	0.37	0.0585	0.339	0.0534	0.3833
20	SLC32A1	rs6027422	0.43	0.0627	0.294	0.0578	0.3338
2	COBLL1	rs7609045	0.35	0.0721	0.210	0.0813	0.1591
16	FTO	rs17817449	0.31	0.0084	0.878	0.0102	0.8513

Chr: chromosome. SNP: single nucleotide polymorphisms. ^1^ Tested SNPs in this population (*n* = 351 men). These SNPs are original SNPs, reported by Kilpeläin et al. [37], or proxies (r^2^ > 0.8). MAF: minor allele frequency. Beta: indicates the regression coefficients per one minor allele. ^2^ Model 1, unadjusted general linear model (GLM). SNPs were tested in an additive model (0, 1, or 2 minor alleles). ^3^ Model 2, GLM adjusted for age. P: *p*-value.

**Table 4:** Association of candidate SNPs with plasma leptin concentrations (ln) in women.

				Unadjusted ^2^			Adjusted ^3^
Chr	Gene	SNP ^1^	MAF	Beta ^2^	P ^2^	Beta ^3^	P ^3^
7	LEP	rs2167289	0.36	−0.0720	0.079	−0.0751	0.067
2	GCKR	rs780093	0.29	−0.0782	0.049	−0.0773	0.052
3	CCNL1	rs17451107	0.37	−0.0853	0.038	−0.0873	0.034
20	SLC32A1	rs6027422	0.43	0.0493	0.245	0.0462	0.277
2	COBLL1	rs7609045	0.35	0.0210	0.619	0.0197	0.640
16	FTO	rs17817449	0.31	0.0078	0.850	0.0108	0.793

Chr: chromosome. SNP: single nucleotide polymorphisms. ^1^ Tested SNPs in this population (*n* = 615 women). These SNPs are original SNPs, reported by Kilpeläin et al. [37], or proxies (r^2^ > 0.8). MAF: minor allele frequency. Beta: indicates the regression coefficients per one minor allele. ^2^ Model 1, unadjusted general linear model (GLM). SNPs were tested in an additive model (0, 1, or 2 minor alleles). ^3^ Model 2, GLM adjusted for age. P: *p*-value.

**Table 5:** Association between SNPs ^1^ in candidate genes (screening) and plasma leptin concentrations (ln) in men and women.

Unadjusted (Model 1)					Adjusted for Sex and Age (Model 2)					Adjusted for Sex, Age, and BMI (Model 3)				
Gene	SNP ^1^	MAF	Beta ^2^	P ^2^	Gene	SNP ^1^	MAF	Beta ^3^	P ^3^	Gene	SNP ^1^	MAF	Beta ^3^	P ^3^
LEPR	rs4567312	0.11	−0.353	0.006	LEPR	rs10749753	0.30	0.080	0.016	LEPR	rs10749753	0.30	0.088	0.001
LEPR	rs2025803	0.21	0.106	0.008	FTO	rs1075440	0.31	−0.089	0.017	LEPR	rs7513047	0.21	0.084	0.002
FTO	rs16952570	0.12	0.256	0.008	LEPR	rs2025803	0.21	0.078	0.017	LEPR	rs9436748	0.23	0.084	0.002
LEPR	rs7513047	0.21	0.106	0.008	LEPR	rs7513047	0.21	0.077	0.019	LEPR	rs2025803	0.21	0.081	0.003
LEPR	rs9436748	0.23	0.104	0.010	COBLL1	rs13410987	0.10	0.112	0.022	LEPR	rs12145690	0.46	−0.066	0.012
FTO	rs7191513	0.45	0.100	0.012	LEPR	rs9436748	0.23	0.076	0.022	COBLL1	rs13410987	0.10	0.100	0.014
COBLL1	rs13410987	0.10	0.147	0.014	FTO	rs17819063	0.04	−0.141	0.031	FTO	rs17819063	0.04	−0.131	0.016
LEP	rs4731427	0.09	−0.188	0.014	FTO	rs9921255	0.17	0.081	0.039	COBLL1	rs355870	0.21	0.079	0.018
COBLL1	rs355849	0.09	0.147	0.016	COBLL1	rs355849	0.09	0.101	0.045	FTO	rs7191513	0.45	0.062	0.022
LEPR	rs10749753	0.30	0.094	0.020	COBLL1	rs7607980	0.11	0.095	0.047	LEPR	rs12409877	0.43	−0.065	0.024
FTO	rs17224310	0.14	0.121	0.020	LEPR	rs3806318	0.18	−0.072	0.048	LEPR	rs17415296	0.10	0.077	0.026
COBLL1	rs7607980	0.11	0.132	0.025						LEPR	rs3806318	0.18	−0.066	0.027
FTO	rs9921255	0.17	0.106	0.028						LEPR	rs9436746	0.43	−0.060	0.029
COBLL1	rs12328675	0.11	0.129	0.029						LEPR	rs10493380	0.12	0.076	0.029
COBLL1	rs10490694	0.11	0.125	0.034						FTO	rs1075440	0.31	−0.065	0.037
LEPR	rs6657868	0.49	−0.089	0.036						LEPR	rs1327115	0.48	−0.058	0.042
LEPR	rs12409877	0.43	−0.087	0.038						LEPR	rs6657868	0.49	−0.059	0.042
COBLL1	rs3769891	0.31	0.093	0.038						LEPR	rs11208675	0.37	−0.062	0.046
LEP	rs7795794	0.06	−0.164	0.041						FTO	rs4784329	0.39	0.057	0.048
COBLL1	rs13017482	0.42	0.081	0.045						COBLL1	rs355911	0.31	0.062	0.048

^1^ SNP: single nucleotide polymorphisms obtained in the whole screening of the corresponding genes as detailed in methods. Only top-ranked SNPs with *p*-value < 0.05 are listed. MAF: minor allele frequency. BMI: body mass index. Beta: indicates the regression coefficients per one minor allele (leptin concentrations are expressed as ln of ng/mL. ^2^ Model 1, unadjusted general linear model (GLM). SNPs were tested in an additive model (0, 1, or 2 minor alleles). ^3^ Model 2, general linear model (GLM) adjusted for sex and age. ^4^ Model 3, Model 2 adjusted for BMI. P: *p*-value.

**Table 6:** Association between SNPs1 in candidate genes (screening) and plasma leptin concentrations (ln) in men.

Adjusted for Age (Model 2)					Adjusted for Age and BMI (Model 3)				
Gene	SNP ^1^	MAF	Beta ^2^	P ^2^	Gene	SNP ^1^	MAF	Beta ^3^	P ^3^
FTO	rs12324955	0.31	0.183	0.005	FTO	rs1075440	0.31	−0.134	0.010
FTO	rs1075440	0.31	−0.162	0.009	FTO	rs741300	0.40	−0.112	0.018
FTO	rs7205009	0.48	0.137	0.012	FTO	rs16952570	0.12	0.283	0.032
FTO	rs6499652	0.34	0.132	0.016	FTO	rs708258	0.48	0.093	0.039
FTO	rs2111115	0.29	0.125	0.023	FTO	rs708262	0.32	−0.102	0.045
FTO	rs9921255	0.17	0.138	0.043					
FTO	rs17819063	0.04	−0.236	0.047					

^1^ SNP: single nucleotide polymorphisms obtained in the whole screening of the corresponding genes as detailed in methods. Only top-ranked SNPs with *p*-value < 0.05 are listed. MAF: minor allele frequency. BMI: body mass index. Beta: indicates the regression coefficients per one minor allele (leptin concentrations are expressed as ln of ng/mL ^2^ Model 2, general linear model (GLM) adjusted for sex and age. SNPs were tested in and additive model (0, 1, or 2 minor alleles). ^3^ Model 3, Model 2 adjusted for BMI. P: *p*-value.

**Table 7:** Association between SNPs^1^ in candidate genes (screening) and plasma leptin concentrations (ln) in women.

Adjusted for Age (Model 2)					Adjusted for Age and BMI (Model 3)				
Gene	SNP ^1^	MAF	Beta ^2^	P ^2^	Gene	SNP ^1^	MAF	Beta ^3^	P ^3^
LEPR	rs12145690	0.46	−0.109	0.004	LEPR	rs12145690	0.46	−0.112	<0.000
LEPR	rs10749753	0.30	0.112	0.007	LEPR	rs3806318	0.18	−0.122	0.001
FTO	rs7186220	0.33	−0.137	0.010	LEPR	rs10749753	0.30	0.111	0.001
LEPR	rs9436297	0.10	0.158	0.010	LEPR	rs9436748	0.23	0.097	0.004
LEPR	rs3806318	0.18	−0.111	0.012	LEPR	rs7513047	0.21	0.095	0.005
LEPR	rs2025803	0.21	0.102	0.013	LEPR	rs2025803	0.21	0.091	0.007
LEPR	rs9436748	0.23	0.101	0.014	FTO	rs17224310	0.14	0.106	0.011
FTO	rs10153154	0.20	0.148	0.014	FTO	rs7191513	0.45	0.084	0.012
LEPR	rs7513047	0.21	0.100	0.015	FTO	rs4784329	0.39	0.089	0.013
FTO	rs4784329	0.39	0.105	0.016	LEPR	rs12409877	0.43	−0.084	0.017
FTO	rs10852525	0.12	0.167	0.017	COBLL1	rs13410987	0.10	0.117	0.017
FTO	rs1362570	0.22	0.179	0.019	FTO	rs7186220	0.33	−0.100	0.023
FTO	rs12931859	0.13	−0.160	0.020	FTO	rs17823199	0.47	0.074	0.025
COBLL1	rs13410987	0.10	0.136	0.022	LEPR	rs12405556	0.32	−0.090	0.025
LEPR	rs970467	0.18	−0.132	0.022	FTO	rs10852525	0.12	0.127	0.028
FTO	rs7194243	0.37	−0.108	0.027	LEPR	rs9436746	0.43	−0.072	0.035
FTO	rs7188162	0.12	0.170	0.028	COBLL1	rs355870	0.21	0.085	0.037
COBLL1	rs355849	0.09	0.130	0.032	GCKR	rs1260326	0.29	−0.068	0.041
FTO	rs11076010	0.10	0.171	0.033	LEPR	rs9436297	0.10	0.104	0.041
FTO	rs9934504	0.23	−0.099	0.042	LEPR	rs1327115	0.48	−0.071	0.042
COBLL1	rs3769891	0.31	0.090	0.043	LEPR	rs1327121	0.38	−0.078	0.044
FTO	rs9806929	0.10	0.152	0.047	LEPR	rs11208654	0.39	−0.077	0.044
GCKR	rs780094	0.30	−0.079	0.047	GCKR	rs2293571	0.37	0.071	0.048
LEPR	rs9436301	0.28	−0.093	0.048					
GCKR	rs1260326	0.29	−0.079	0.049					

^1^ SNP: single nucleotide polymorphisms obtained in the whole screening of the corresponding genes as detailed in methods. Only top-ranked SNPs with *p*-value < 0.05 are listed. MAF: minor allele frequency. BMI: body mass index. Beta: indicates the regression coefficients per one minor allele (leptin concentrations are expressed as ln of ng/mL ^2^ Model 2, general linear model (GLM) adjusted for sex and age. SNPs were tested in and additive model (0, 1, or 2 minor alleles). ^3^ Model 3, Model 2 adjusted for BMI. P: *p*-value.

**Table 8:** GWAS results for the association between the top-ranked SNPs and plasma leptin concentrations (Ln) adjusted for sex and age in the whole population.

Adjusted for Sex and Age (Model 2)					
Gene	SNP ^1^	Chr	MAF	Beta ^2^	P ^2^
LOC105378641	rs10737381	1	0.32	−0.160	1.51 × 10^−^^6^
CHN2	rs245908	7	0.38	−0.160	1.66 × 10^−^^6^
LOC105378641	rs6425900	1	0.32	−0.157	2.02 × 10^−^^6^
__	rs7218921	17	0.44	−0.151	2.34 × 10^−^^6^
TP63	rs1515495	3	0.13	−0.276	3.12 × 10^−^^6^
MTHFS	rs2562744	15	0.41	−0.146	5.73 × 10^−^^6^
GPR15	rs4857399	3	0.20	−0.209	6.18 × 10^−^^6^
__	rs4937802	11	0.43	0.161	6.75 × 10^−^^6^
__	rs9271170	6	0.24	0.161	7.56 × 10^−^^6^
__	rs2218396	19	0.25	0.158	1.01 × 10^−^^5^
LOC105379315	rs17093306	8	0.13	−0.192	1.04 × 10^−^^5^
__	rs1533357	14	0.18	−0.239	1.07 × 10^−^^5^
LOC105378641	rs1418483	1	0.41	0.147	1.14 × 10^−^^5^
__	rs12422977	12	0.20	−0.155	1.18 × 10^−^^5^
DISC1FP1	rs1507608	11	0.14	0.165	1.18 × 10^−^^5^
LOC105378641	rs12136079	1	0.48	0.147	1.19 × 10^−^^5^
CNTNAP2	rs10259411	7	0.28	−0.149	1.26 × 10^−^^5^
MARCH2	rs11667516	19	0.06	−0.223	1.47 × 10^−^^5^
DISC1FP1	rs7104915	11	0.13	0.164	1.84 × 10^−^^5^
CPNE4	rs3914906	3	0.14	0.209	1.93 × 10^−^^5^
__	rs4494315	11	0.35	0.136	2.07 × 10^−^^5^
CNTNAP2	rs6464830	7	0.30	−0.145	2.18 × 10^−^^5^
SAMD5	rs6936905	6	0.09	0.249	2.35 × 10^−^^5^
SNTB1	rs4297067	8	0.25	−0.190	2.42 × 10^−^^5^
SMYD3	rs6693295	1	0.32	−0.177	2.51 × 10^−^^5^
PITPNC1	rs12937353	17	0.48	−0.146	2.51 × 10^−^^5^
LOC105378316	rs10763548	10	0.23	0.139	2.56 × 10^−^^5^
__	rs4418823	11	0.45	0.143	2.56 × 10^−^^5^
CNTNAP2	rs6954225	7	0.28	−0.144	2.61 × 10^−^^5^
SMYD3	rs10924513	1	0.31	−0.211	2.75 × 10^−^^5^
MTHFS	rs6495451	15	0.48	−0.135	2.87 × 10^−^^5^
__	rs4704594	5	0.27	0.138	2.95 × 10^−^^5^
UBASH3B	rs10790525	11	0.45	0.135	3.00 × 10^−^^5^

^1^ SNP: single nucleotide polymorphisms. Only top-ranked SNPs with *p*-value < 3 × 10^−5^ for the whole population (*n* = 966) are listed. MAF: minor allele frequency. BMI: body mass index. Chr: chromosome. Beta: indicates the regression coefficients per one minor allele (leptin concentrations are expressed as ln of ng/mL). ^2^ Model 2, general linear model (GLM) adjusted for sex and age. P: *p*-value.

**Table 9:** GWAs results for the Gene–sex interactions between the top-ranked SNPs and sex in determining plasma leptin concentrations (ln) in the whole population.

	Men		Women		P-Gene*sex			
SNP	Beta ^1^	SE ^1^	Beta ^2^	SE ^2^	Interaction ^3^	MAF	Chr	Gene
rs11954861	0.623	0.158	−0.532	0.125	1.01 × 10^−8^	0.12	5	SLIT3
rs1146714	−0.235	0.067	0.144	0.049	5.21 × 10^−6^	0.30	1	intergenic
rs1407608	0.288	0.071	−0.102	0.050	7.26 × 10^−6^	0.17	13	LINC01048
rs10082903	−0.229	0.061	0.101	0.043	8.68 × 10^−6^	0.41	12	LOC105370084
rs1984722	−0.511	0.131	0.182	0.085	9.01 × 10^−6^	0.20	17	ABCA9
rs7511972	−0.442	0.129	0.259	0.099	1.73 × 10^−5^	0.06	1	DAB1
rs16970458	0.136	0.057	−0.167	0.042	1.80 × 10^−5^	0.42	15	BUB1B-PAK6
rs3754886	−0.162	0.061	0.161	0.045	1.89 × 10^−5^	0.26	2	INPP4A
rs6753380	−0.160	0.061	0.161	0.045	1.95 × 10^−5^	0.30	2	INPP4A
rs1857745	−0.181	0.070	0.184	0.050	1.99 × 10^−5^	0.26	2	__
rs17680173	0.205	0.055	−0.082	0.039	2.20 × 10^−5^	0.42	4	__
rs1343093	−0.205	0.067	0.147	0.049	2.29 × 10^−5^	0.30	1	__
rs3754891	−0.160	0.061	0.158	0.045	2.42 × 10^−5^	0.33	2	INPP4A
rs1777888	−0.206	0.068	0.146	0.049	2.43 × 10^−5^	0.30	1	__
rs1552098	0.290	0.091	−0.162	0.058	2.95 × 10^−5^	0.30	15	ADAMTS17

^1^ SNP: single nucleotide polymorphisms. Only top-ranked SNPs with *p*-value < 3 × 10^−5^ for the whole population (*n* = 966) are listed. Beta: indicates the regression coefficients per one minor allele (leptin concentrations are expressed as ln of ng/mL). SE: standard error. MAF: minor allele frequency. Chr: chromosome. Beta^1^: indicates the regression coefficients for men (*n* = 351). Beta^2^: indicates the regression coefficients for women (*n* = 615). ^3^ *p*-value obtained for the interaction term SNP*sex in the corresponding hierarchical GLM regression model including the main effects.

## Supplementary Materials

The following are available online at https://www.mdpi.com/2072-6643/11/11/2751/s1: Supplemental Table S1: Linkage disequilibrium (LD) plots (r^2^ values) for the statistically significant SNPs in the: (a) FTO and (b) LEPR genes in the whole population (n=966); Supplemental Table S2: Association of candidate SNPs with plasma leptin concentrations (ln), additionally adjusted for BMI and stratified by men and women; Supplemental Table S3: Gene*Sex interactions between the SNPs in candidate genes (screening) in determining plasma Leptin concentrations (Ln) in men and women; Supplemental Table S4: GWAS results for the association between the top-ranked SNPs and plasma leptin concentrations (Ln) adjusted for sex, age and BMI in the whole population.; Supplemental Figure S1: Association of candidate SNPs with plasma leptin concentrations (Ln) additionally adjusted for BMI in men and women; Supplemental Figure S2: Q–Q plot for the GWAS on plasma leptin concentrations in the whole population; Supplemental Table S5 GWAS results for the association between the top-ranked SNPs and plasma leptin concentrations (Ln) in men; Supplemental Figure S3: Regional plot for the top-ranked SNP rs10737381 located at LOC105378641, on chromosome 1. Results for the whole population; Supplemental Figure S4: Dotplot for plasma leptin concentrations (ln) depending on the CHN2 genotypes; Supplemental Figure S5: Regional plot for the top-ranked SNP rs11954861 located at SLIT3, on chromosome 5. Results for the gene*interaction in the whole population; Supplemental Figure S6. Regional plot for the top-ranked SNP rs1146714 (intergenic), on chromosome 1. Results for the gene*interaction in the whole population; Supplemental Table S6: GWAS results for the association between the top-ranked SNPs and plasma leptin concentrations (Ln) in women.
