# Supplementary material for: Candidate Gene and Genome-Wide Association Studies for Circulating Leptin Levels Reveal Population and Sex-Specific Associations in High Cardiovascular Risk Mediterranean Subjects
Source: Nutrients. 2019 Nov 13;11(11):2751. doi: 10.3390/nu11112751 (PMC6893551; doi:10.3390/nu11112751)
Supplement: Supplementary file 1 [file nutrients-11-02751-s001.pdf]

ONLINE SUPPORTING MATERIAL

Candidate gene and genome-wide association studies for circulating leptin levels reveal population and sex-specific associations in high cardiovascular risk Mediterranean subjects

Carolina Ortega-Azorín, Oscar Coltell, Eva M. Asensio, Jose V. Sorlí, José I. González, Olga Portolés, Carmen Saiz, Ramon Estruch, Judith B Ramírez-Sabio, Alejandro Pérez-Fidalgo, Jose M Ordovás and Dolores Corella

Supplemental Table 1. 2
Supplemental Table 2. 2
Supplemental Table 3 3
Supplemental Figure 1 4
Supplemental Figure 2. 5
Supplemental Table 4 6
Supplemental Figure 3 7
Supplemental Figure 4 8
Supplemental Figure 5 9
Supplemental Figure 6 10
Supplemental Table 5 11
Supplemental Table 6 12

Supplemental Table 1. Association of candidate SNPs with plasma Leptin concentrations (ln) additionally adjusted for BMI in men and women.

Chr	Gene	SNP1	SNP/Proxy[2]	Beta[3]	P[3]
7	LEP	rs10487505	rs2167289	-0.008	0.771
2	GCKR	rs780093	rs780093	-0.029	0.286
3	CCNL1	rs900400	rs17451107	-0.069	0.016
20	SLC32A1	rs6071166	rs6027422	0.023	0.434
2	COBLL1	rs6738627	rs7609045	0.029	0.305
16	FTO	rs8043757	rs17817449	0.007	0.805

Chr: Chromosome.

[1] Single Nucleotide Polymorphism (SNP)s reported by Kilpeläin et al [37].

[2] Tested SNPs in this population (n=966 men and women). These SNPs are the original SNPs or proxies ($r^2 > 0.8$).

[3] Model 3, GLM adjusted for sex, age and BMI. Beta: Indicates the regression coefficients per one minor allele.

Supplemental Table 2. Association of candidate SNPs with plasma Leptin concentrations (ln), additionally adjusted for BMI and stratified by men and women.

			Men		Women	
Chr	Gene	SNP[1]	Beta[2]	P[2]	Beta[3]	P[3]
7	LEP	rs2167289	0.046	0.331	-0.043	0.202
2	GCKR	rs780093	0.021	0.652	-0.058	0.077
3	CCNL1	rs17451107	0.014	0.791	-0.111	0.001
20	SLC32A1	rs6027422	0.044	0.379	0.012	0.741
2	COBLL1	rs7609045	0.049	0.315	0.018	0.609
16	FTO	rs17817449	0.019	0.671	-0.001	0.984

Chr: Chromosome.

[1] Single Nucleotide Polymorphism (SNP)s reported by Kilpeläin et al [37], or proxies ($r^2 > 0.8$) for this population (n=351 men and 615 women).

[2] Model 1, unadjusted general lineal model (GLM). SNPs were tested in and additive model (0, 1 or 2 minor alleles).

[3] Model 2, GLM adjusted for sex and age. Beta: Indicates the regression coefficients per one minor allele.

Supplemental Table 3. Gene*Sex interactions between the SNPs in candidate genes (screening) in determining plasma Leptin concentrations (ln) in men and women.

Gene	SNP[1]	MAF	P-interaction SNP*sex[2]	Men		Women	
				Beta[3]	SE[3]	Beta[4]	SE[4]
LEPR	rs9436297	0.10	0.003	-0.153	0.084	0.158	0.061
FTO	rs1362570	0.22	0.010	-0.157	0.107	0.182	0.076
FTO	rs10852525	0.12	0.011	-0.131	0.093	0.165	0.070
FTO	rs7194243	0.37	0.014	0.093	0.066	-0.109	0.048
FTO	rs7205009	0.48	0.015	0.138	0.055	-0.028	0.041
FTO	rs7188162	0.12	0.016	-0.153	0.109	0.170	0.077
FTO	rs6499652	0.34	0.017	0.132	0.055	-0.030	0.041
FTO	rs12324955	0.31	0.019	0.189	0.064	0.005	0.046
FTO	rs11076010	0.10	0.021	-0.149	0.112	0.168	0.080
FTO	rs12931859	0.13	0.022	0.124	0.103	-0.158	0.069
FTO	rs2111115	0.29	0.025	0.125	0.055	-0.027	0.041
FTO	rs9806929	0.10	0.026	-0.146	0.109	0.149	0.076
LEPR	rs12405556	0.32	0.027	0.086	0.064	-0.092	0.049
FTO	rs7186220	0.33	0.027	0.057	0.072	-0.140	0.053
LEPR	rs12145690	0.46	0.033	0.033	0.056	-0.111	0.038
LEP	rs2278815	0.46	0.036	-0.104	0.055	0.039	0.040
FTO	rs708262	0.32	0.037	-0.110	0.060	0.044	0.042
FTO	rs11646488	0.17	0.041	0.124	0.085	-0.092	0.062

[1] SNP: Single Nucleotide Polymorphisms. Only top-ranked SNPs with P-value < 0.05 are listed. MAF: Minor allele frequency. BMI: Body Mass Index. Beta: Indicates the regression coefficients per one minor allele (leptin concentrations are expressed as ln of ng/mL). [2] *P-value* obtained for the interaction term SNP*Sex in the corresponding hierarchical GLM regression model including the main effects. Beta[3]: indicates the regression coefficients for men (n=351). Beta[4]: indicates the regression coefficients for women (n=615).

Supplemental Figure 1. Linkage disequilibrium (LD) plots (r^2 values) for the statistically significant SNPs in the: (a) FTO and (b) LEPR genes in the whole population (n=966).

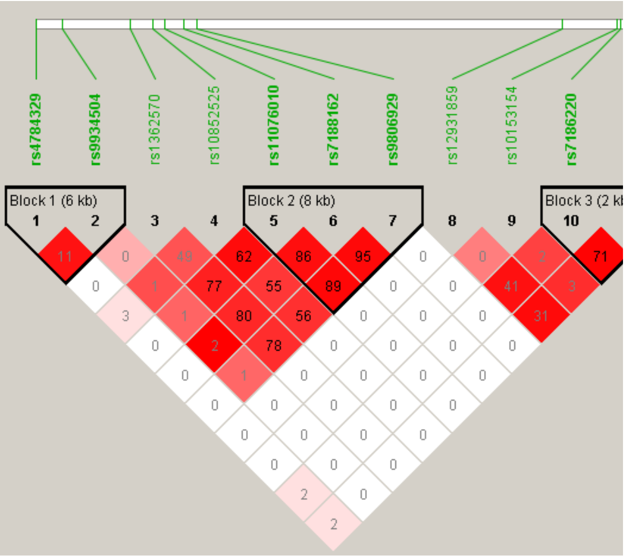

(**a**)

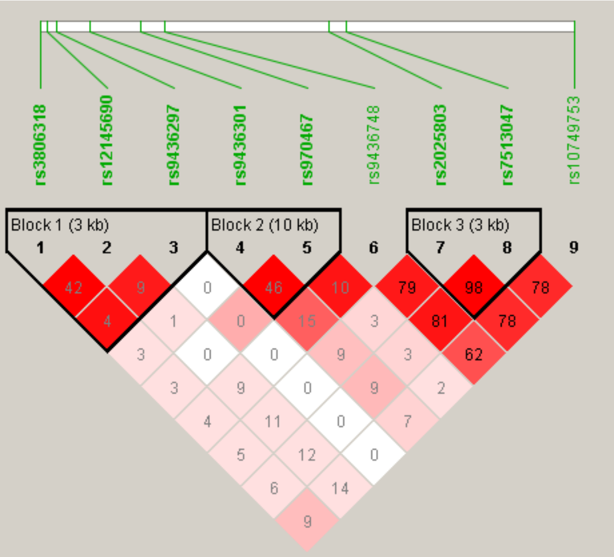

(**b**)

Supplemental Figure 2. Q-Q plot for the GWAS on plasma leptin concentrations in the whole population (n=966).

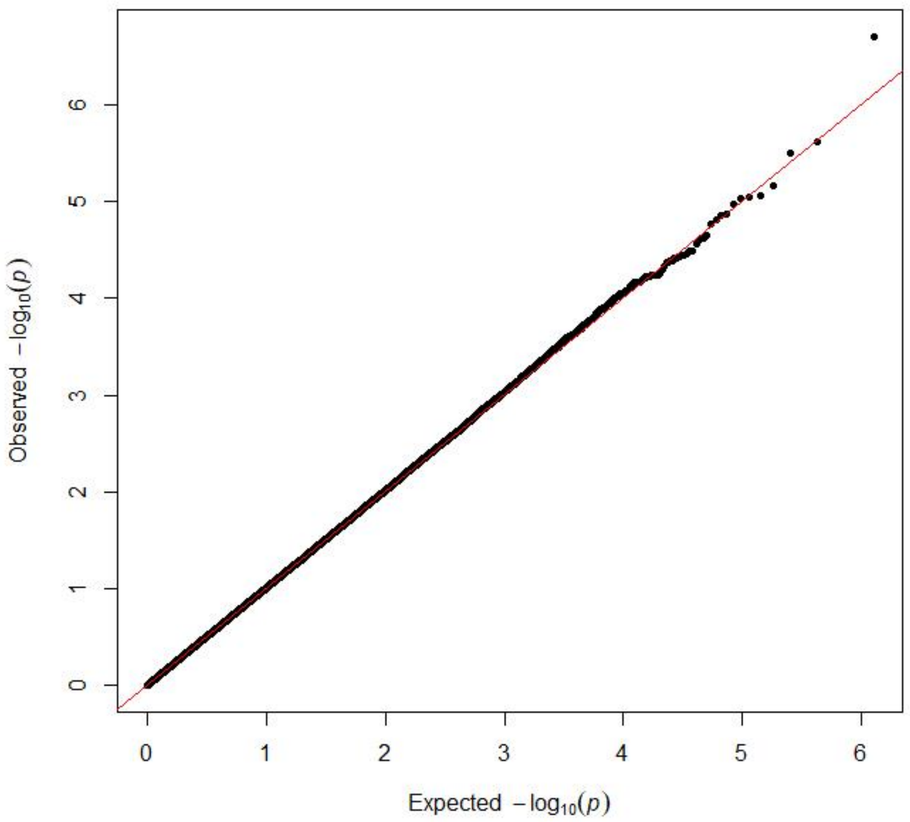

Supplemental Table 4. GWAS results for the association between the top-ranked SNPs and plasma leptin concentrations (ln) adjusted for sex, age and BMI in the whole population.

Adjusted for sex, age and BMI (model 3)

Gene	SNP[1]	MAF	Beta[2]	*P*[2]
__	rs2218396	0.25	0.137	4.14E-06
ADGB	rs9390422	0.40	-0.127	7.98E-06
SMYD3	rs10924513	0.31	-0.182	1.42E-05
AES	rs367825	0.34	-0.127	2.07E-05
__	rs6811915	0.15	-0.133	2.78E-05
__	rs6437472	0.25	0.123	3.31E-05
__	rs7218921	0.44	-0.110	3.41E-05
CNTNAP2	rs10268597	0.46	0.114	3.56E-05
LOC105378641	rs10737381	0.32	-0.114	3.69E-05
__	rs717061	0.06	-0.187	3.70E-05
__	rs2061345	0.25	0.122	3.81E-05
GPR15	rs4857399	0.20	-0.158	4.15E-05

[1] Single Nucleotide Polymorphisms. Only top-ranked SNPs with *P-value* < 0.05 are listed. MAF: Minor allele frequency. BMI: Body Mass Index. Beta: Indicates the regression coefficients per one minor allele (leptin concentrations are expressed as ln of ng/mL).
[2] Model 3, general lineal model (GLM). adjusted for sex, age and BMI.

Supplemental Figure 3. Regional plot for the top-ranked SNP rs10737381 located at LOC105378641, on chromosome 1. Results for the whole population.

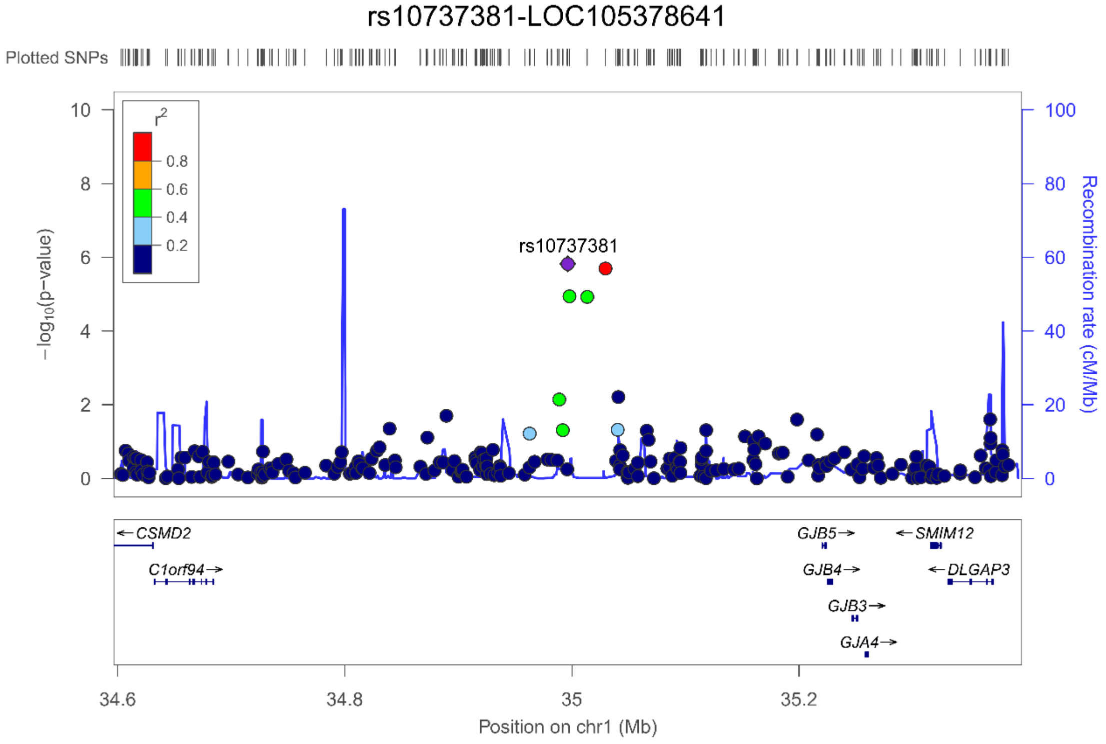

Supplemental Figure 4. Dotplot for plasma leptin concentrations (ln) depending on the CHN2 genotypes.

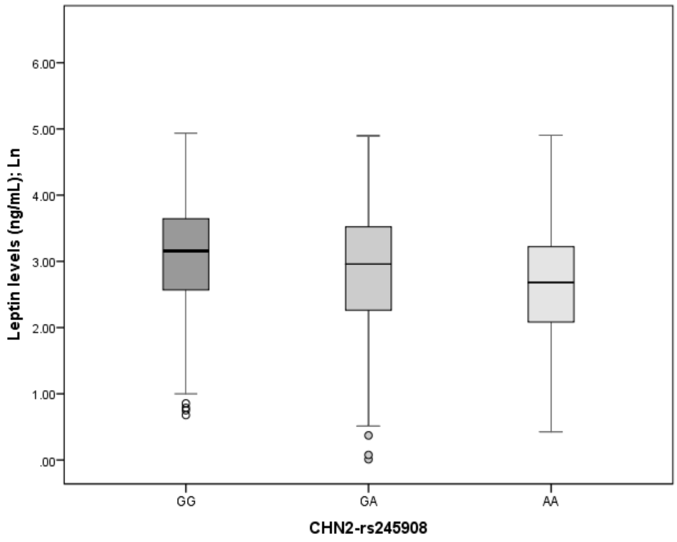

Associations between the rs245908- CHN2 SNP (n= 382 GG, 444 GA and 138 AA) and plasma leptin concentrations (ln) in the whole population (unadjusted values; P = 1.95x10-7 for differences between genotypes).

Supplemental Figure 5. Regional plot for the top-ranked SNP rs11954861 located at SLIT3, on chromosome 5. Results for the gene*interaction in the whole population.

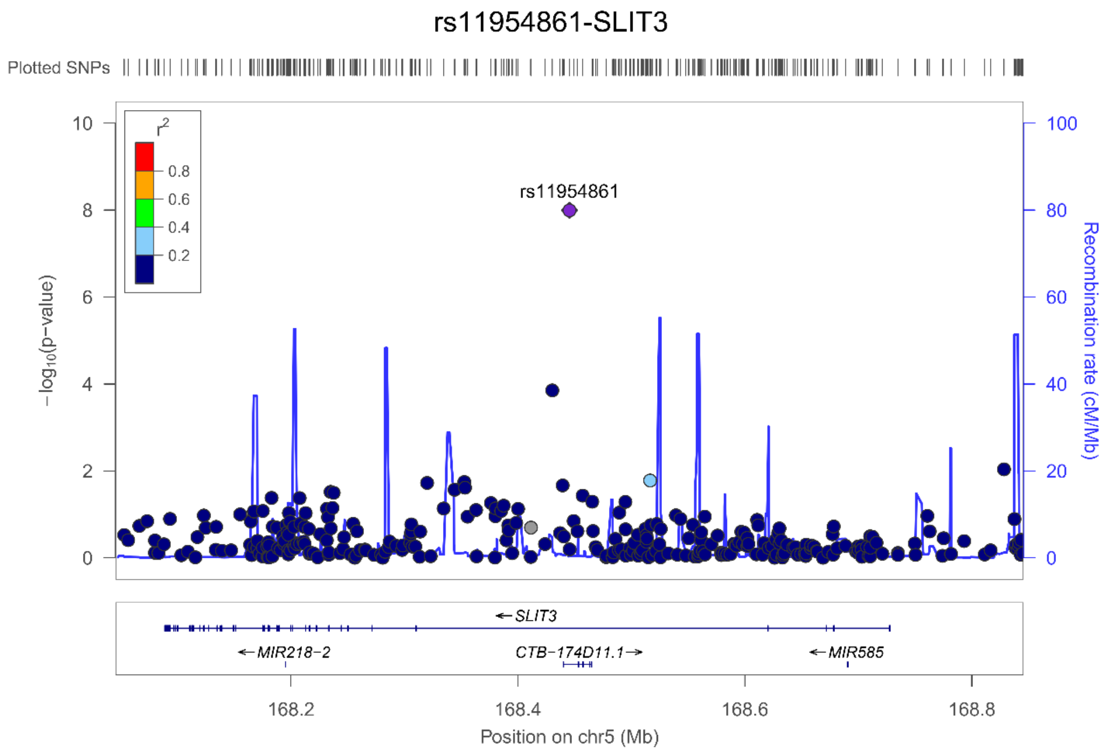

Supplemental Figure 6. Regional plot for the top-ranked SNP rs1146714 (intergenic), on chromosome 1. Results for the gene*interaction in the whole population.

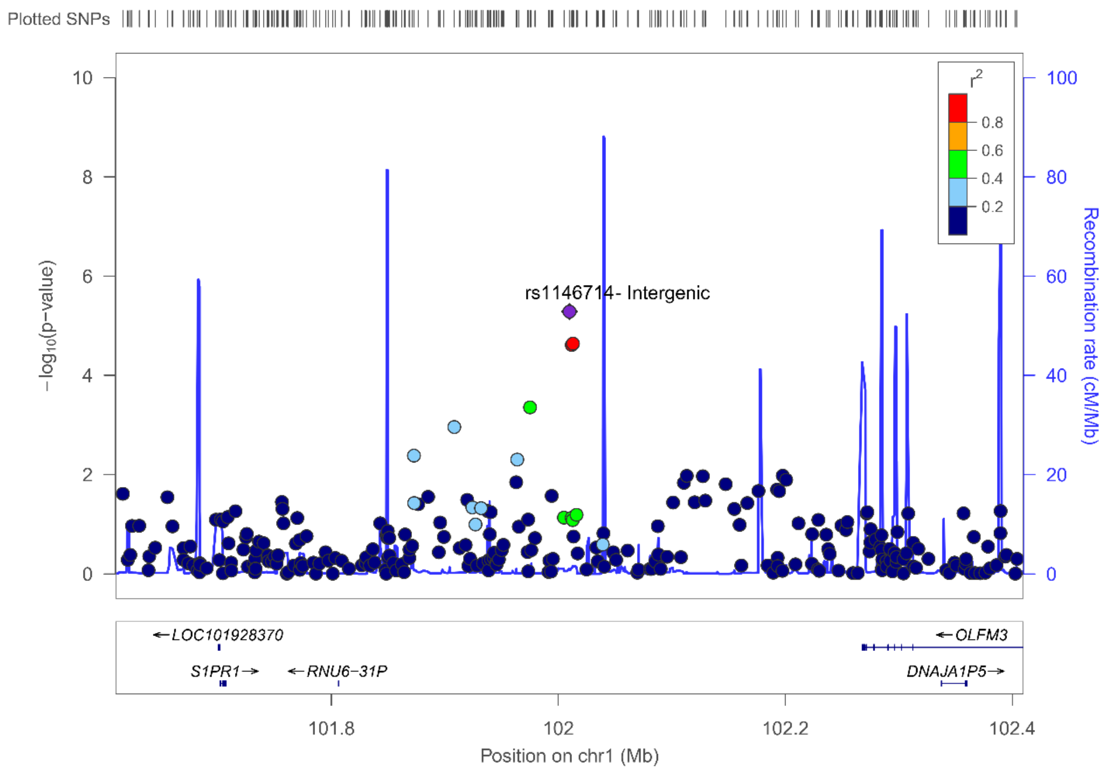

Supplemental Table 5. GWAS results for the association between the top-ranked SNPs and plasma leptin concentrations (ln) in men.

Unadjusted (model 1)					Adjusted for sex and age (model 2)					Adjusted for sex, age and BMI (model 3)				
Gene	SNP[1]	MAF	Beta[2]	*P*[2]	Gene	SNP[1]	MAF	Beta[3]	*P*[3]	Gene	SNP[1]	MAF	Beta[4]	*P*[4]
__	rs4074110	0.37	0.276	3.28E-06	__	rs4074110	0.37	0.272	4.48E-06	EEF1DP3	rs4141377	0.17	-0.246	9.83E-06
__	rs7218921	0.44	-0.240	6.10E-06	SORCS1	rs607437	0.44	0.249	5.63E-06	__	rs12747311	0.04	-0.262	1.30E-05
SORCS1	rs607437	0.44	0.244	8.63E-06	__	rs5755145	0.20	0.277	8.89E-06	NTRK2	rs4361832	0.14	-0.256	1.31E-05
__	rs5755145	0.20	0.277	9.05E-06	__	rs7218921	0.44	-0.235	1.06E-05	__	rs9841498	0.48	0.217	1.50E-05
LOC105373013	rs738256	0.22	0.267	1.91E-05	SORCS1	rs685316	0.50	-0.231	1.71E-05	MIR217HG	rs1368242	0.22	-0.226	2.24E-05
LOC105376387	rs10795522	0.33	0.274	1.97E-05	LOC105376387	rs10795522	0.33	0.275	1.85E-05	__	rs4074110	0.37	0.212	2.33E-05
__	rs4915725	0.08	-0.479	2.07E-05	__	rs10961505	0.11	-0.282	2.16E-05	EEF1DP3	rs365	0.18	-0.236	2.62E-05
__	rs9841498	0.48	0.252	2.50E-05	__	rs4915725	0.08	-0.477	2.16E-05	EEF1DP3	rs4942034	0.17	-0.237	2.64E-05
__	rs10961505	0.11	-0.279	2.59E-05	LOC105373013	rs738256	0.22	0.265	2.20E-05	__	rs2163643	0.06	0.604	2.81E-05
DLG2	rs7933597	0.09	0.358	2.81E-05	SORCS1	rs669061	0.48	-0.227	2.34E-05	__	rs717061	0.06	-0.333	3.13E-05
SORCS1	rs685316	0.50	-0.223	3.09E-05	__	rs9841498	0.48	0.250	2.76E-05	CORO2B	rs11633874	0.39	-0.191	3.45E-05
NCF4	rs4821544	0.35	0.241	3.22E-05	DLG2	rs7933597	0.09	0.358	2.77E-05	__	rs1416261	0.29	0.195	3.62E-05
__	rs717061	0.06	-0.395	3.25E-05	NCF4	rs4821544	0.35	0.239	3.87E-05	GLP2R	rs4239110	0.29	-0.370	3.66E-05
FAM65B	rs9379703	0.30	-0.274	3.52E-05	__	rs9481736	0.13	-0.716	4.02E-05	NTRK2	rs10868241	0.32	-0.215	3.74E-05
__	rs6009945	0.40	0.235	3.75E-05	SNTB1	rs4297067	0.25	-0.323	4.17E-05	__	rs982422	0.39	0.258	4.01E-05
BTBD7	rs1263429	0.12	0.284	4.12E-05	__	rs717061	0.06	-0.388	4.39E-05	CACNA1B	rs7852364	0.26	0.527	4.31E-05
LOC105373013	rs5755183	0.23	0.256	4.17E-05	BTBD7	rs1263429	0.12	0.282	4.42E-05	__	rs8055091	0.45	0.193	4.80E-05
__	rs9481736	0.13	-0.716	4.21E-05	LOC105373013	rs5755183	0.23	0.255	4.45E-05					
SORCS1	rs669061	0.48	-0.218	4.79E-05	LINC01048	rs1407608	0.17	0.292	4.62E-05					

[1] Single Nucleotide Polymorphisms for this population (n=351). Only top-ranked SNPs with *P-value* < 5×10^{-5} are listed. MAF: Minor allele frequency. BMI: Body Mass Index. Beta: Indicates the regression coefficients per one minor allele (leptin concentrations expressed as ln of ng/mL). [2] Model 1, unadjusted general lineal model (GLM). SNPs were tested in and additive model (0, 1 or 2 minor alleles). [3] Model 2, general lineal model (GLM) adjusted for sex and age. [4] Model 3, Model 2 adjusted for BMI.

Supplemental Table 6. GWAS results for the association between the top-ranked SNPs and plasma leptin concentrations (ln) in women.

Unadjusted (model 1)					Adjusted for sex and age (model 2)					Adjusted for sex, age and BMI (model 3)				
Gene	SNP[1]	MAF	Beta[2]	*P*[2]	Gene	SNP[1]	MAF	Beta[3]	*P*[3]	Gene	SNP[1]	MAF	Beta[4]	*P*[4]
CPNE4	rs3914906	0.14	0.318	9.04E-08	CPNE4	rs3914906	0.14	0.316	1.19E-07	SMOC2	rs12530037	0.37	-0.156	1.86E-06
GPR15	rs4857399	0.20	-0.270	2.71E-06	GPR15	rs4857399	0.20	-0.269	3.07E-06	SLIT3	rs11954861	0.12	-0.494	2.05E-06
LOC105378316	rs10763548	0.23	0.186	3.22E-06	LOC105378316	rs10763548	0.23	0.183	4.84E-06	ELOA-AS1	rs11591202	0.22	-0.160	4.51E-06
__	rs4937802	0.43	0.201	5.87E-06	__	rs4937802	0.43	0.200	6.40E-06	DNAH8	rs9394554	0.16	0.175	5.69E-06
__	rs11663063	0.04	-0.400	9.37E-06	__	rs10749415	0.13	-0.421	9.35E-06	ADGB	rs9390422	0.40	-0.156	6.56E-06
SPACA9	rs2231400	0.12	-0.377	9.79E-06	SPACA9	rs2231400	0.12	-0.374	1.13E-05	DNAH8	rs9349103	0.17	0.162	7.23E-06
PSORS1C3	rs9468877	0.16	0.250	1.09E-05	__	rs11663063	0.04	-0.396	1.18E-05	HMGCL	rs2076343	0.21	-0.152	1.35E-05
LOC105378653	rs523864	0.14	0.211	1.24E-05	PSORS1C3	rs9468877	0.16	0.248	1.30E-05	DNAH8	rs9366986	0.09	0.214	1.62E-05
__	rs1533357	0.18	-0.288	1.29E-05	__	rs1533357	0.18	-0.288	1.32E-05	MROH5	rs2748414	0.37	0.140	2.04E-05
__	rs10749415	0.13	-0.413	1.34E-05	LOC105378653	rs523864	0.14	0.208	1.64E-05	MROH5	rs2748421	0.37	0.141	2.24E-05
MATN2	rs4735521	0.35	-0.197	1.41E-05	DOCK8	rs4741867	0.50	-0.173	1.76E-05	SGK1	rs9376026	0.48	-0.142	2.25E-05
__	rs11752823	0.31	-0.222	1.55E-05	__	rs10090034	0.20	-0.364	1.78E-05	UBASH3B	rs4935810	0.42	0.149	2.39E-05
TTI1	rs6068552	0.25	-0.234	1.94E-05	MATN2	rs4735521	0.35	-0.195	1.79E-05	PRR16	rs17429498	0.18	0.143	2.68E-05
__	rs4494315	0.35	0.170	2.00E-05	__	rs4494315	0.35	0.170	2.01E-05	SLIT3	rs10055309	0.09	-0.291	2.75E-05
RPRD1B	rs6022547	0.28	-0.233	2.08E-05	AGBL1	rs7170517	0.35	-0.173	2.11E-05	LOC101927630	rs13221764	0.47	0.139	2.79E-05
PALM2	rs2795058	0.23	-0.197	2.10E-05	__	rs11752823	0.31	-0.219	2.32E-05	DNAH8	rs9394555	0.32	0.141	3.00E-05
DOCK8	rs4741867	0.50	-0.171	2.11E-05	TTI1	rs6068552	0.25	-0.231	2.49E-05	CUL2	rs12184386	0.32	-0.140	3.14E-05
__	rs10090034	0.20	-0.361	2.12E-05	UBASH3B	rs10892893	0.28	-0.170	2.68E-05	PRR16	rs12187844	0.25	0.140	3.61E-05
SLIT3	rs11954861	0.12	-0.532	2.54E-05	RPRD1B	rs6022547	0.28	-0.230	2.68E-05	__	rs1146714	0.30	0.165	3.88E-05
UBASH3B	rs10790525	0.45	0.171	2.70E-05	UBASH3B	rs10790525	0.45	0.171	2.77E-05	PRR16	rs2122193	0.21	0.135	3.91E-05
AGBL1	rs7170517	0.35	-0.169	3.00E-05	PALM2	rs2795058	0.23	-0.194	2.83E-05	HMGCL	rs2473375	0.36	-0.138	4.06E-05
__	rs28477158	0.06	-0.453	3.24E-05	SLIT3	rs11954861	0.12	-0.529	2.83E-05	C5orf66	rs2652093	0.31	0.173	4.28E-05
LOC105378316	rs6481397	0.35	0.162	3.25E-05	C5orf66	rs2652093	0.31	0.214	2.85E-05	LOC105372980	rs999223	0.20	-0.153	4.34E-05

LOC105378316	rs1427213	0.48	-0.173	3.29E-05	__	rs4418823	0.45	0.174	3.70E-05	ERBB2	rs4252596	0.07	0.225	4.39E-05
C5orf66	rs2652093	0.31	0.213	3.31E-05	SLC15A1	rs4646211	0.11	-0.293	3.80E-05	HMGCL	rs2179395	0.34	-0.138	4.73E-05
__	rs10742524	0.09	-0.447	3.39E-05	__	rs28477158	0.06	-0.448	4.03E-05					
UBASH3B	rs10892893	0.28	-0.167	3.64E-05	__	rs7463498	0.27	-0.339	4.08E-05					
__	rs4418823	0.45	0.174	3.69E-05	__	rs5768403	0.22	-0.182	4.09E-05					
__	rs5768403	0.22	-0.183	3.82E-05	TMIGD3	rs10776732	0.21	-0.248	4.16E-05					
SLC15A1	rs4646211	0.11	-0.293	3.85E-05	LOC105378316	rs1427213	0.48	-0.171	4.21E-05					
LOC401478	rs1398034	0.40	-0.219	3.90E-05	__	rs10742524	0.09	-0.441	4.40E-05					
__	rs10113535	0.06	-0.431	4.20E-05	LOC105378316	rs6481397	0.35	0.160	4.47E-05					
L3MBTL3	rs6569648	0.10	-0.197	4.56E-05	DNAH8	rs9394554	0.16	0.190	4.68E-05					
__	rs1496236	0.21	-0.285	4.57E-05	LOC401478	rs1398034	0.40	-0.216	4.85E-05					
__	rs7463498	0.27	-0.336	4.76E-05	PKNOX2	rs10893331	0.46	-0.180	4.98E-05					
DNAH8	rs9394554	0.16	0.189	4.97E-05										

[1] Single Nucleotide Polymorphisms for this population (n=615). Only top-ranked SNPs with *P-value* < 5.00×10^{-5} are listed. MAF: Minor allele frequency. BMI: Body Mass Index. Beta: Indicates the regression coefficients per one minor allele (leptin concentrations expressed as ln of ng/mL). [2] Model 1, unadjusted general lineal model (GLM). SNPs were tested in and additive model (0, 1 or 2 minor alleles). [3] Model 2, general lineal model (GLM) adjusted for sex and age. [4] Model 3, Model 2 adjusted for BMI.
